# Sequential Delivery of Host-Induced Virulence Effectors by Appressoria and Intracellular Hyphae of the Phytopathogen *Colletotrichum higginsianum*

**DOI:** 10.1371/journal.ppat.1002643

**Published:** 2012-04-05

**Authors:** Jochen Kleemann, Linda J. Rincon-Rivera, Hiroyuki Takahara, Ulla Neumann, Emiel Ver Loren van Themaat, H. Charlotte van der Does, Stéphane Hacquard, Kurt Stüber, Isa Will, Wolfgang Schmalenbach, Elmon Schmelzer, Richard J. O'Connell

**Affiliations:** 1 Department of Plant-Microbe Interactions, Max-Planck-Institute for Plant Breeding Research, Cologne, Germany; 2 Central Microscopy Max-Planck-Institute for Plant Breeding Research, Cologne, Germany; 3 Max Planck Genome Centre Cologne, Cologne, Germany; University of Melbourne, Australia

## Abstract

Phytopathogens secrete effector proteins to manipulate their hosts for effective colonization. Hemibiotrophic fungi must maintain host viability during initial biotrophic growth and elicit host death for subsequent necrotrophic growth. To identify effectors mediating these opposing processes, we deeply sequenced the transcriptome of *Colletotrichum higginsianum* infecting *Arabidopsis*. Most effector genes are host-induced and expressed in consecutive waves associated with pathogenic transitions, indicating distinct effector suites are deployed at each stage. Using fluorescent protein tagging and transmission electron microscopy-immunogold labelling, we found effectors localised to stage-specific compartments at the host-pathogen interface. In particular, we show effectors are focally secreted from appressorial penetration pores before host invasion, revealing new levels of functional complexity for this fungal organ. Furthermore, we demonstrate that antagonistic effectors either induce or suppress plant cell death. Based on these results we conclude that hemibiotrophy in *Colletotrichum* is orchestrated through the coordinated expression of antagonistic effectors supporting either cell viability or cell death.

## Author Summary

Many fungal plant pathogens undergo a series of developmental and morphological transitions required for successful host invasion. For example, *Colletotrichum higginsianum*, a pathogen of cruciferous plants, employs a two-stage infection strategy called ‘hemibiotrophy’: after specialized penetration organs (appressoria) breach the host cuticle and cell wall, the fungus initially produces bulbous primary hyphae inside living epidermal cells (‘biotrophy’), before entering a destructive phase in which host tissues are killed and macerated by filamentous secondary hyphae (‘necrotrophy’). Here we investigated the role of secreted effector proteins in mediating hemibiotrophy and their delivery at fungal-plant interfaces. We found expression of many effector genes is plant-induced and distinct sets of effectors are deployed in successive waves by particular fungal cell-types. Early-expressed effector proteins are focally secreted from appressorial penetration pores and may function to suppress early plant defense responses, which we found to be activated before fungal entry. We also show that later-expressed effectors accumulate in structures formed at the interface between primary hyphae and living host cells, implicating these hyphae in effector delivery. Our findings indicate new functions for fungal infection structures and suggest a model for how this fungus switches from biotrophy to necrotrophy.

## Introduction

To penetrate the cuticle and cell wall of their hosts, most plant pathogenic fungi differentiate specialized infection structures called appressoria. Appressoria have been long-recognized as providing tight adhesion to host surfaces (Latin *appressus*, pressed closely against) [1]. The appressoria of *Colletotrichum* and *Magnaporthe* species display a complex physiology and morphology, adapted for efficient host cell entry. Key features are (a) a melanized cell wall acting as a semipermeable barrier to osmolytes, (b) glycerol accumulation for generating turgor and (c) an extracellular matrix to anchor the cell and counter-balance downward mechanical forces applied during penetration [2]. The appressoria of *Colletotrichum* and *Magnaporthe* are highly polarized cells with an upper domed region and a basal region containing the penetration pore, from which a needle-like penetration hypha emerges to puncture the epidermal cell wall [3], [4]. Differentiation of the pore involves deposition of a new wall layer, termed the ‘pore wall overlay’, which is continuous with the penetration hypha cell wall [5]. Hydrolytic enzymes secreted by penetration hyphae may act synergistically with mechanical pressure during host penetration [2]. However, whether appressoria actively manipulate the attacked cell in preparation for invasion is currently unknown.

Host manipulation and re-programming are hallmarks of biotrophic plant pathogens, which depend on living host cells. In addition to overcoming preformed barriers, these pathogens must defeat immune responses elicited by recognition of conserved microbe-associated molecular patterns (MAMPS, e.g. chitin), including local deposition of chemical and physical barriers at pathogen entry sites [6]–[8]. Since MAMPs fulfill important functions in pathogens and cannot be modified or jettisoned without fitness cost, biotrophic pathogens secrete effector proteins as molecular weapons to evade or suppress plant immunity. The evolution of secreted effector proteins by pathogens led plants in turn to evolve resistance proteins that recognize these effectors, thereby providing effector-triggered immunity, often leading to host cell death and pathogen arrest. In turn, pathogens deploy effectors to interfere with these processes, resulting in a molecular arms-race between plant and pathogen in which both opponents try to overcome each others innovations, leaving only temporary winners [9]. Pathogen effectors often carry the marks of this rapid co-evolution, showing extreme sequence diversification. Effectors typically have no similarity to known proteins and have a restricted phylogenetic distribution [10].

*Colletotrichum* species are notorious plant pathogens, most of which have a ‘hemibiotrophic’ lifestyle that combines an initial, symptomless biotrophic phase with a later necrotrophic phase associated with severe symptoms. In contrast to biotrophs, *Colletotrichum* species can be cultured axenically and are accessible to genetic manipulation [11]. *C. higginsianum* has a wide host range, including many cruciferous crops and the model plant *Arabidopsis thaliana*. Phylogenetically, *C. higginsianum* belongs to the *C. destructivum* species group, which is characterized by ‘localized biotrophy’, where intracellular biotrophic hyphae are restricted to the first-infected epidermal cell [12]. Filamentous necrotrophic hyphae later develop from the bulbous biotrophic hyphae and spread into the surrounding tissue, producing macerated, water-soaked lesions [13]. Little is known about *Colletotrichum* effectors: Stephenson and co-workers [14] reported CgDN3, a putative secreted protein of *C. gloeosporioides* which was implicated in suppressing host resistance responses. Furthermore, *C. lindemuthianum* and *C. higginsianum* possess CIH1 [15], [16], an effector containing tandem chitin-binding lysin motifs which may function in chitin sequestration and camouflage [10].

We assume that the appressoria, penetration hyphae and biotrophic hyphae of *C. higginsianum* secrete effector proteins before, during and after penetration to evade host defenses and maintain host viability during the biotrophic phase, and to induce cell death at the switch to necrotrophy. In the present study, we aimed to investigate the role of secreted effector proteins in mediating hemibiotrophy and their delivery at fungal-plant interfaces. Based on deep transcriptome sequencing and computational mining of ESTs from precise infection stages, we derived a large inventory of *in planta*-expressed effector candidates for this pathogen. Tagged effectors were found to localize to previously undescribed interfacial compartments. In particular, we demonstrate that effectors are focally secreted from appressorial penetration pores before host invasion. Furthermore, we present evidence that the coordinated expression and secretion of antagonistic biotrophy effectors and toxin effectors contribute to fungal virulence and the regulation of hemibiotrophy in *C. higginsianum*.

## Results

### Deep transcriptome sequencing uncovers a large repertoire of *in planta*-expressed effectors

As a first step towards the discovery of secreted *C. higginsianum* effector proteins, we generated ESTs by sequencing the fungal transcriptome associated with different cell types and infection stages. These included plant-penetrating appressoria, mature biotrophic hyphae isolated from *Arabidopsis* leaves by fluorescence-activated cell sorting and the late necrotrophic phase. Sequencing techniques, strategies used to maximize gene discovery as well as EST assembly statistics are summarized in Text S1. Biocomputational screening yielded 327 EST contigs predicted to encode solubly secreted, extracellular proteins. We defined *C. higginsianum* effector candidates (ChECs) as secreted proteins lacking homologs outside the genus *Colletotrichum* or resembling (presumed) effectors from other plant pathogenic fungi. Applying these criteria, 198 contigs encoding ChECs were identified, of which 102 were depleted in ESTs derived from the late necrotrophic phase (Table S1). Thus, these genes appear to be preferentially expressed during infection stages relevant to the establishment and maintenance of biotrophy, namely appressoria and biotrophic hyphae, and we refer to them as biotrophy-associated ChECs hereafter. Most of these were small in size (average 67, median 56 amino acids) and lacked recognizable protein domains. A motif search revealed no motifs were shared by non-paralogous ChECs.

Only 30% of the biotrophy-associated ChECs had a detectable homolog in the closely-related species *C. graminicola*, suggesting most ChECs are *higginsianum*-specific ‘orphan’ genes. In contrast, among an equal number of similar-sized genes randomly selected from the genome, 59% had *C. graminicola* homologs, indicating that biotrophy-associated ChEC genes are subject to greater diversification than other genes. Consistent with this, a survey of 21 different *Colletotrichum* species and isolates by Southern analysis showed that *ChEC1* and *ChEC2* were strongly conserved within the *C. destructivum* species group, and ChEC3 was only detectable in *C. higginsianum* isolates (Figure S1A). ChEC3 and its paralog ChEC3a are similar to the *C. gloeosporioides* effector CgDN3 [14], and lack homologs in *C. graminicola*. ChEC3, ChEC3a and CgDN3 are small proteins (47 to 56 amino acids after signal peptide cleavage), and have only 17 residues in common. Despite that, their exon-intron structure and predicted secondary structure are conserved (Figure S1B). Sequencing *ChEC3* and *ChEC3a* loci from 20 different *C. higginsianum* isolates revealed that *ChEC3* is monoallelic and *ChEC3a* has an additional allele with only one (nonsynonymous) nucleotide polymorphism. Thus, both genes show interspecies diversification (or absence) but intraspecies conservation.

Five other ChECs displayed sequence similarity to effectors previously identified from other fungi. ChEC5 harbours a cerato-platanin domain and shares 79% identical amino acids with *M. oryzae* MSP1 [17]. Both, ChEC90 and ChEC90a contain LysM domains and are homologous to *C. lindemuthianum* CIH1 [15] 39% and 50% amino acid identity, respectively). ChEC36 shares 40% identical amino acids with *Fusarium oxysporum* f.sp. *lycopersici* SECRETED IN XYLEM 6 (SIX6) [18] while ChEC88 resembles the biotrophy-associated secreted protein BAS3 of *M. oryzae* [19] (49% amino acid identity). Remarkably, a survey of the top 30 contigs containing the highest numbers of ESTs from plant-penetrating appressoria, revealed that no fewer than 18 (60%) encode secreted proteins, of which 12 were biotrophy-associated ChECs (Table S2). In addition to ChEC3 and its paralog ChEC3a, these included ChEC4 and ChEC9, both predicted to contain nuclear localization signals. The functionality of these signals was experimentally verified by transient expression *in planta* (Figure S2), raising the possiblity that ChEC4 and ChEC9 are translocated into the host nucleus for transcriptional reprogramming. Interestingly, ChEC7 and ChEC10 had transcripts containing remnants of retrotransposons within their UTRs, which resembled CgT1, a non-LTR LINE-like element previously identified in *C. gloeosporioides* [20] and Ccret2 from *C. cereale* [21], respectively ([22]; Table S2). Taken together, secreted proteins, including ChECs, predominate among the most highly expressed genes in appressoria during early host invasion.

**Figure 1:**
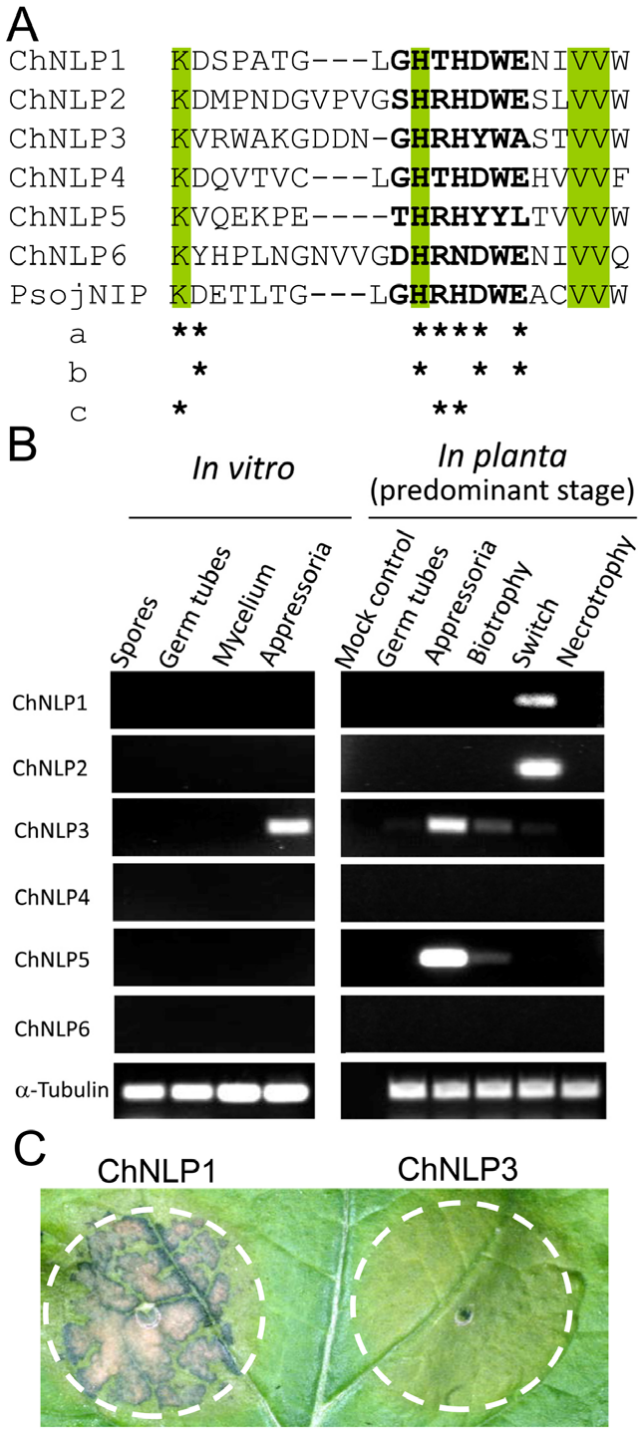
The *C. higginsianum* genome contains six members of the Necrosis- and ethylene inducing peptide 1-like protein (NLP) gene family with contrasting expression profiles and necrosis-inducing activities. (A) The genome of *C. higginisianum* harbours six members of the *NLP* family: *ChNLP1* is the most similar (5e^−44^) and *ChNLP6* the least similar homolog (3e^−9^, possibly truncated) of PsojNIP from *Phytophathora sojae* [25]. Shown is an alignment encompassing the conserved NLP consensus motif “GHRHDWE” of ChNLP1-6 and PsojNIP. Conserved amino acid residues are shaded green. Asterisks indicate residues crucial for NLP activity: a, amino acid residues investigated by Ottmann and co-workers [37] by alanine replacements resulting in abolished (b) or reduced (c) activity. ChNLP3 and ChNLP5 lacked three of these crucial residues, while ChNLP6 lacked one. In contrast, ChNLP1, ChNLP2 and ChNLP4 contained all crucial residues. (B) Detection of *ChNLP* transcripts in different fungal cell types *in vitro* and during plant infection. *ChNLP1* and *ChNLP2* were exclusively expressed during the switch from biotrophy to necrotrophy. Neither *ChNLP4* nor *ChNLP6* were expressed in the sampled material. *ChNLP3* and *ChNLP5* were strongly upregulated in appressoria penetrating host cells, with transcripts still detectable during biotrophy. *ChNLP5*, but not *ChNLP3*, appeared plant-induced as indicated by absence of transcripts in *in vitro* appressoria. α-tubulin was used as reference to allow for variation of fungal biomass. (C) Transient expression of full-length ChNLP1 or ChNLP3 in *Nicotiana benthamiana* leaves. Pictures were taken 6 days after agroinfiltration. ChNLP1 causes severe necrosis, whereas ChNLP3 does not, as expected from the sequence alignment and expression pattern.

In addition to ChECs that may support the biotrophic lifestyle, we identified putative secreted toxin effectors, including ChToxB, a homolog of the host-selective toxin ToxB from *Pyrenophora tritici-repentis* [23] and homologs of Necrosis- and Ethylene-inducing Peptide1-Like Proteins (referred to as NLPs hereafter) [24]. The six NLP homologs identified in the *C. higginsianum* genome show sequence variation in the NLP consensus motif and have contrasting expression profiles and necrosis-inducing activities. For example, ChNLP1 is expressed specifically at the switch to necrotrophy and is a potent cell death inducer when expressed transiently in *Nicotiana benthamiana*, whereas ChNLP3 is expressed in appressoria before penetration and lacks necrosis-inducing activity (Figure 1).

### Successive waves of effector gene expression accompany pathogenic transitions

**Figure 2:**
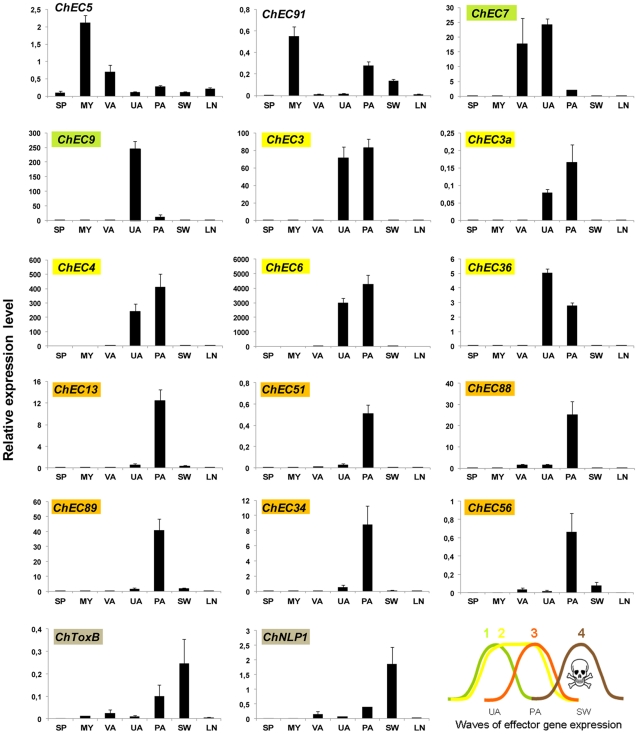
Expression profiling of selected biotrophy-associated ChEC and putative toxin genes by qRT-PCR. Expression levels are shown relative to the mean expression of actin and α-tubulin. Genes with highest expression *in planta* are highlighted with colours, indicating distinct waves of effector gene expression. *In vitro* cell types are: dormant spores (SP), saprotrophic mycelium (MY) and mature appressoria (VA). *In planta* stages are: unpenetrated appressoria (UA), penetrated appressoria with nascent biotrophic hyphae beneath (PA), biotrophy to necrotrophy switch (SW) and late necrotrophy (LN).

The sampling of biological materials used for EST generation was designed to maximize the discovery of genes expressed at several biotrophy-relevant stages (from unpenetrated appressoria through to very mature biotrophic hyphae) and did not allow dissection of gene expression dynamics associated with developmental transitions (e.g. pre-/post-invasive growth and the switch from biotrophy to necrotrophy). To profile the expression of selected ChECs and putative toxins during infection in more detail, we used qRT-PCR. We sampled RNA from the following developmental stages: unpenetrated appressoria *in planta*, penetrated appressoria with nascent biotrophic hyphae, the switch from biotrophy to necrotrophy (Figure S3) as well as late necrotrophy. To represent *in vitro* cell types, dormant spores, saprotrophic mycelium and mature appressoria formed on an artificial, non-penetratable substratum were included. For expression profiling, we prioritized ChECs that resembled previously identified effectors (see above) and/or displayed high expression levels in biotrophy-relevant stages as determined by their EST read counts. Expression analysis of 17 selected genes revealed that four successive waves of effector gene expression occur during pathogenesis (Figure 2): The first wave of ChEC genes is induced in unpenetrated appressoria *in planta*, exemplified by *ChEC7* and *ChEC9*. Similarly, *ChEC3*, *ChEC3a*, *ChEC4*, *ChEC6* and *ChEC36* are also induced in unpenetrated appressoria *in planta*, but their expression continues into the early biotrophic phase (wave two). Of these, ChEC6 had the highest relative expression level of all ChECs tested, suggesting an important role in early pathogenesis. In contrast, *ChEC13*, *ChEC34*, *ChEC51*, *ChEC56*, *ChEC88* and *ChEC89* are specifically induced during penetration and establishment of biotrophic hyphae (wave three). The last wave of effector genes, exemplified by *ChNLP1* and *ChToxB*, is induced only during the switch to necrotrophy, suggesting that their toxic products contribute to terminating the biotrophic phase for subsequent necrotrophic growth. In contrast to the previous examples, *ChEC5* and *ChEC91* were preferentially induced in saprotrophic mycelium. Thus, nearly all ChECs tested were confirmed to be biotrophy-associated. Moreover, for seven *ChEC* genes showing induction in unpenetrated appressoria *in planta* (*ChEC7, 9, 3, 3a, 4, 6, 36*), pre-formed transcripts were not detectable in dormant spores and only *ChEC7* was induced in mature appressoria *in vitro*, indicating all other genes are truly plant-induced.

### Appressorial penetration pores as a nanoscale interface for focal ChEC delivery

To localize ChECs during pathogenesis, proteins were expressed in *C. higginsianum* as C-terminal fusions with fluorescent proteins under control of their native upstream regulatory sequences. At least three independent transformants were analyzed per gene and verified to show the same localization pattern. Live-cell confocal laser scanning microscopy detected fluorescence for all of the seven ChECs tested (see below), confirming the computational ORF predictions. For high-resolution localization of selected protein fusions we also used transmission electron microscopy-immunocytochemistry with an mRFP-specific antibody to label ultrathin sections.

**Figure 3:**
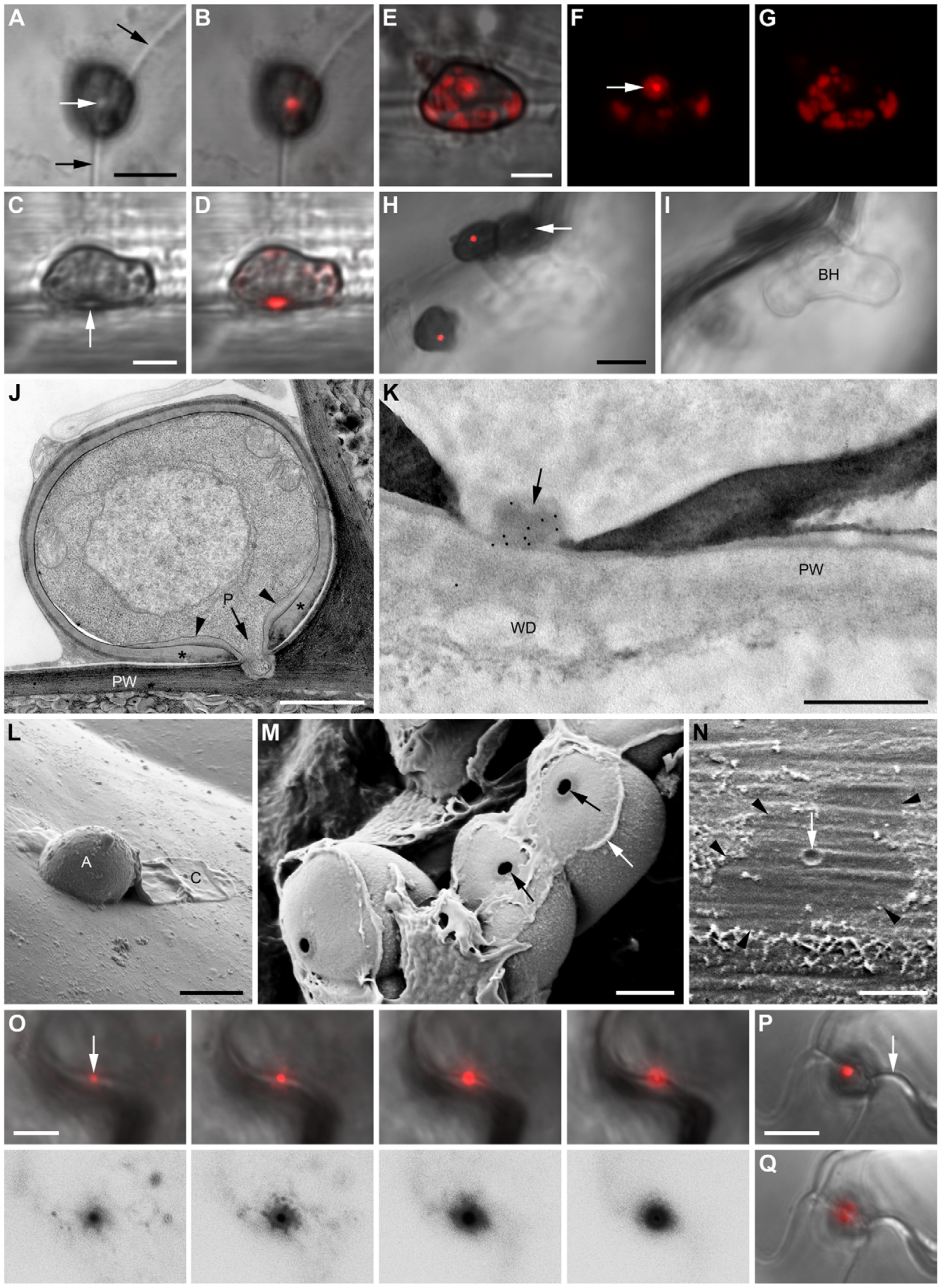
Appressorial pores as an interface for focal ChEC delivery. Transformant appressoria expressing the wave 2 effectors ChEC36:mRFP (A–O) and ChEC6:mRFP (P, Q). Appressoria or penetration sites after removal of appressoria were examined by confocal laser scanning microscopy viewed from above (A, B, E–I, O–Q) or from the side (C, D), and with transmission electron microscopy (J, K) and scanning electron microscopy (L–N). (A–D) Bright field and maximum fluorescence intensity overlay images of appressoria. Black arrows indicate the anticlinal plant cell wall and white arrows the penetration pore. (E) Fluorescence overlay image of an appressorium showing weak peripheral labelling of intracellular structures. (F, G) Fluorescence recorded with identical settings at the base (F) and the center (G) of the appressorium shown in (E). Arrow indicates a fluorescent ring surrounding the brightly fluorescing pore. (H, I) Fluorescence overlays recorded with identical settings focused on appressorial pores (H) or biotrophic hyphae (BH) formed beneath a penetrated appressorium (arrow). (J) Median section through an appressorium viewed with transmission electron microscopy (fixed with glutaraldehyde-osmium tetroxide and embedded in epoxy resin). A penetration hypha evaginates from the pore (P). An additional layer of the appressorial wall (asterisk) forms a thickened ring (arrowheads) around the pore, continuous with the penetration hypha wall. PW, plant cell wall. (K) Immunogold labelling of an appressorial pore (arrow) using antibodies recognizing mRFP (cells fixed in formaldehyde-glutaraldehyde and embedded in acrylic resin). PW, plant cell wall. WD, host cell wall deposits. (L) Scanning electron microscope image showing attached turgid appressorium (A) and collapsed conidium (C) on a leaf surface. (M) Plant-exposed underside of detached appressoria with penetration pores (black arrows) and remnants of extracellular matrix and/or plant cuticle (white arrow). (N, O) Penetration sites from which appressoria were detached completely. (N) The lobed outline of a former appressorium is still visible (arrowheads) with a mark representing the penetration point (arrow). (O) Micrograph series representing different focal planes as fluorescence overlay (top panels) and corresponding black on white conversion of the fluorescence channel (bottom panels), focusing from the penetration point (left) downwards into the plant cell wall (right). Arrow: inserted penetration hypha. (P, Q) Fluorescence overlays focused on the appressorial pore (P) and the underlying plant cell wall (Q). Arrow, anticlinal plant cell wall. Images were recorded at 24 hours post inoculation (hpi) (A–G, K, P, Q), 32 hpi (J, L–O), 40 hpi (H, I). Scale bars: 5 µm (A, H, L, N, O, P) and 2 µm (C, E, M), 1 µm (J), 500 nm (K). See also Figure S4.

When fungal transformants expressing ChEC36:mRFP (a wave 2 effector) were inoculated onto *Arabidopsis* seedlings, we detected a strongly fluorescent spot at the basal penetration pore in 72% of the inspected appressoria (n = 101) (Figure 3A–D). In some cases, the pore was in addition encircled by a weakly fluorescent ring (Figure 3E, F). Among pore-labelled appressoria, 11% showed in addition labelling of discrete intracellular structures in the fungal cytoplasm (Figure 3E, G). Transmission electron microscopy immunogold labelling revealed that these structures resembled vacuolar inclusion bodies (Figure S4A). Biotrophic hyphae showed no labelling (Figure 3H, I; Figure S4B), suggesting ChEC36:mRFP is exclusively secreted before and during penetration. Transmission electron microscopy of *in planta* appressoria revealed that penetration pores (∼200 nm diameter) are surrounded by an additional wall layer continuous with the penetration hypha wall (Figure 3J), referred to as the ‘pore wall overlay’ hereafter, which contains β-1,3-glucan (Figure S5). Based on serial sectioning of 24 appressoria, immunogold labelling confirmed that ChEC36:mRFP specifically localized to the penetration pore in 13 appressoria (54%) and decorated the pore wall overlay in seven appressoria (29%) (Figure 3K, Figure S4C, D). No labelling was observed on the inner or outer surface of the appressorial wall, suggesting that appressoria secrete ChECs in a highly polarized manner towards the pore.

Membrane contrast was low in these samples because they were not fixed with osmium tetroxide and were embedded in acrylic resin in order to provide optimal antigen preservation. Nevertheless, in favourably-orientated sections, the immunogold labelling of pores and pore wall overlays appeared external to the fungal plasma membrane, consistent with ChEC36 being an extracellular protein (Figure S4E). No immunogold labelling was observed in or beneath wild-type appressoria (n = 11), indicating specific epitope recognition (Figure S4F). All appressoria with labelled pores had not produced a visible penetration hypha, as verified by serial sectioning. Despite that, small pads of host cell wall material were already deposited beneath 77% of inspected unpenetrated appressoria (n = 35), suggesting host cells respond to the pathogen prior to any visible ingression or structural damage (Figure 3K; Figure S4C, F; Figure S5H). The inner, first-formed layer of these host cell wall deposits did not contain detectable callose but subsequently became encrusted with a callose layer (Figure S5H).

Appressoria *in situ* can be acetone-fixed and completely detached from the plant surface by cellulose acetate stripping, as determined by scanning electron microscopy (Figure 3L–N). This allowed us to dissect whether fluorescent protein-tagged ChECs are released from appressoria into the plant epidermis. When the cellulose acetate-stripped leaf surface was inspected with confocal microscopy, sites of successful penetration were characterized by brightly fluorescent spots from which the fluorescence signal appeared to diffuse laterally a short distance (∼2 µm), resulting in a small halo of mRFP fluorescence (Figure 3O). Similar to ChEC36:mRFP, 72% (n = 101) of intact appressoria expressing ChEC6:mRFP (another wave 2 effector) also showed pore-localized fluorescence, with haloes visible at lower focal planes, consistent with local delivery of ChEC6 into the plant apoplast (Figure 3P, Q). Taken together, it appears that ChECs expressed pre-penetration are focally secreted to, and from, an extremely localized zone of direct contact between host and pathogen, delimited by the appressorial penetration pore.

### ChEC delivery to the intimate biotrophic interface between host and pathogen

**Figure 4:**
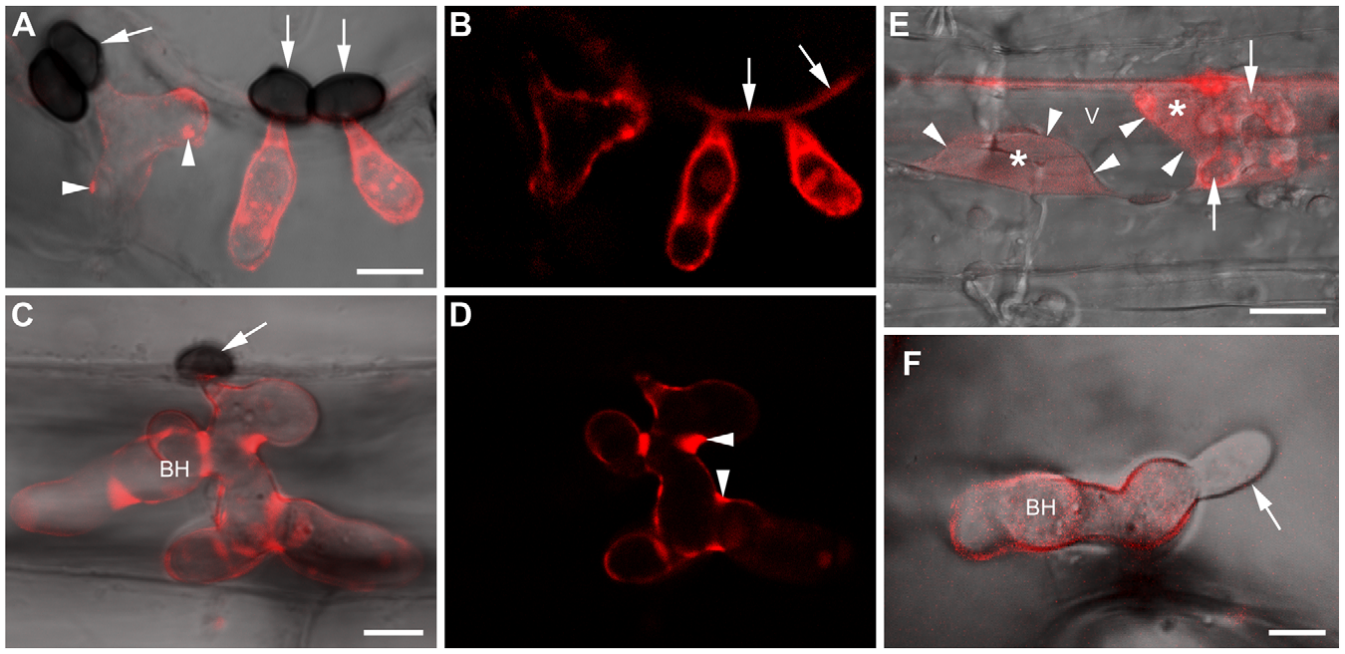
ChEC delivery to the biotrophic interface and host apoplast. Transformant biotrophic hyphae expressing CHEC89:mRFP (wave 3 effector) viewed with confocal laser scanning microscopy. (A) Maximum fluorescence intensity overlay projection of appressoria (arrows) and underlying biotrophic hyphae showing fluorescent foci (arrowheads) on the hyphal surface. (B) Single optical section from (A) showing labelling of the plant cell wall (arrows). (C, D) Mature biotrophic hypha, viewed as in (A and B), showing fluorescence accumulation in hyphal concavities (arrowheads). Arrow: appressorium. (E) Epidermal cell infected by a biotrophic hypha (arrows) showing fluorescence in the apoplastic space (*) enlarged by plasmolysis. Arrowheads demarcate the host plasma membrane. V, vacuole of the host protoplast. See Figure S7 for the corresponding bright field image. (F) Unlabelled necrotrophic hypha (arrow) emerging from a biotrophic hypha. Images were recorded at 43 hpi (A–E) and 55 hpi (F). Scale bars: 10 µm (E) and 5 µm (A, C, F). BH, biotrophic hypha. See also Figure S6.

Inoculation of transformants expressing CHEC89:GFP or CHEC89:mRFP (a wave 3 effector) onto *Arabidopsis* seedlings showed fluorescent labelling on the surface of 91% (n = 89) biotrophic hyphae. In an independent quantification of labelled hyphae, 61% (n = 239) showed punctate accumulation of fluorescence in discrete foci randomly scattered over the hyphal surface (Figure 4A, B; Figure S6A–C). Fully-expanded, mature biotrophic hyphae also showed strong surface labelling, which accumulated in hyphal concavities (Figure 4C, D). After retraction of the plant protoplast by plasmolysis, a fluorescent signal was detectable in the enlarged apoplastic space, suggesting that the CHEC89:mRFP fusion protein is freely diffusible and not linked to the fungal cell wall (Figure 4E, Figure S7). In support of this, we could detect fluorescence in anticlinal plant cell walls near infection sites, especially where two or more neighboring appressoria had invaded the same epidermal cell (Figure 4B; Figure S6C). This suggests the interface between the host plasma membrane and biotrophic hyphae is continuous with the bulk apoplast, allowing limited diffusion away from the penetration site. Spectral scanning confirmed an mRFP-specific fluorescence emission, ruling out local autofluorescence of the plant cell wall. Secondary hyphae emerging from the apices of biotrophic hyphae lacked detectable labelling, indicating CHEC89:mRFP secretion is specific to biotrophic hyphae (Figure 4F).

**Figure 5:**
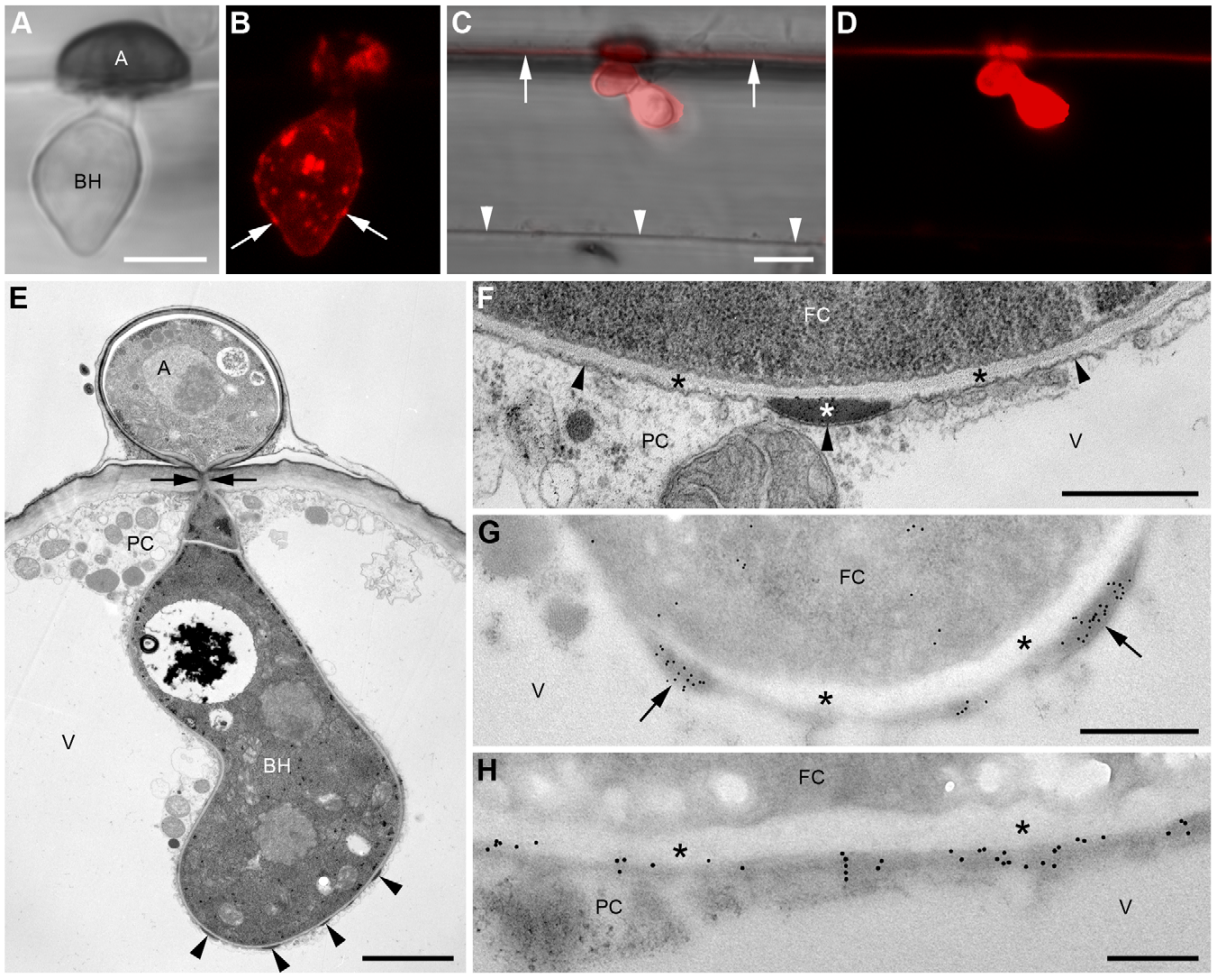
ChECs accumulate in interfacial bodies and diffuse into the host cell wall. Transformant biotrophic hyphae expressing ChEC34:mRFP (wave 3 effector). (A, B) Bright field micrograph and corresponding maximum fluorescence intensity projection. Arrows: fluorescent foci. (C, D) Biotrophic hypha expressing CHEC34:mRFP viewed with confocal laser scanning microscopy settings optimized to show fluorescence in the penetrated epidermal cell wall (arrows). Arrowheads: unpenetrated wall of the same cell. (E, F) Transmission electron microscopy of a wild-type appressorium that produced a biotrophic hypha underneath with interfacial bodies (arrowheads). Arrows indicate the penetration site of the host cell wall. Cells were fixed with glutaraldehyde-osmium tetroxide and embedded in epoxy resin. (F) Close-up of an interfacial body (white asterisk) located between the plant plasma membrane (black arrowheads) and the fungal cell wall (black asterisk). (G, H) Immunogold cytochemistry using an antibody recognizing mRFP labels (G) interfacial bodies (arrows) or (H) the plant-fungal interface. Cells were fixed in formaldehyde-glutaraldehyde and embedded in acrylic resin. A, appressorium. FC, fungal cytoplasm. PC, plant cytoplasm. V, plant vacuole. BH, biotrophic hypha. (*) Fungal cell wall. Images were recorded at 40 hpi (A, B) and 43 hpi (C–H). Scale bars: 5 µm (A, C), 2.5 µm (E), 500 nm (F, G) and 250 nm (H).

Similar to CHEC89:mRFP, localization to the surface of biotrophic hyphae was also observed for the wave 2 effector ChEC3:mRFP (92%, n = 98) and the wave 3 effectors CHEC13:mRFP (86%, n = 114) and CHEC34:mRFP (89%, n = 102) (Figure 5A, B; Figure S6D–I). However, the localization patterns of these ChECs were not identical: Thus, in independent quantification experiments, many hyphae expressing ChEC34:mRFP (75%, n = 140) showed an accumulation of fluorescence in discrete foci. In contrast, the proportion of hyphae showing punctate labelling was lower for transformants expressing ChEC3:mRFP (13%, n = 117) and ChEC13:mRFP (13%, n = 109) which both displayed a more uniform labelling on the surface of most hyphae. Epidermal cells infected by biotrophic hyphae expressing ChEC3:mRFP also showed weak fluorescence in the apoplastic space enlarged by plasmolysis (Figure S6I). Only CHEC13:mRFP was detectable in ‘pseudo biotrophic hyphae’ formed after penetration of an artificial, penetratable substratum, suggesting the expression of all other *CHEC* genes tested is plant-induced and not linked to appressorial penetration *per se* (Figure S8). In contrast to ChEC3:mRFP and CHEC13:mRFP, CHEC34:mRFP also localized to the plant cell wall. This signal was confined to cell walls adjoining penetration sites but spread longer distances (>25 µm) than CHEC89:mRFP (Figure 5C, D).

Using transmission electron microscopy to view the biotrophic interface at higher resolution, we found that the host plasma membrane made direct contact with the cell walls of biotrophic hyphae, except in small regions where discrete pads of electron-opaque material protruded from the hyphal surface (Figure 5E, F). To investigate whether these interfacial bodies correspond to the punctate fluorescence observed by confocal microscopy, we used immunogold labelling to detect CHEC34:mRFP. All interfacial bodies we examined were intensely labelled in transformant (n = 11), but not wild-type biotrophic hyphae (n = 8) (Figure S9), suggesting they are foci of effector accumulation (Figure 5G). In other hyphae where interfacial bodies were not visible, gold labelling more uniformly decorated the plant-fungal interface (Figure 5H). Neither the fungal cell wall nor plant cytoplasm were labelled in these samples. Taken together, our findings are consistent with *Colletotrichum* biotrophic hyphae having a role in effector delivery.

### Effectors antagonizing plant cell death and supporting multiplication of plant pathogenic bacteria

**Figure 6:**
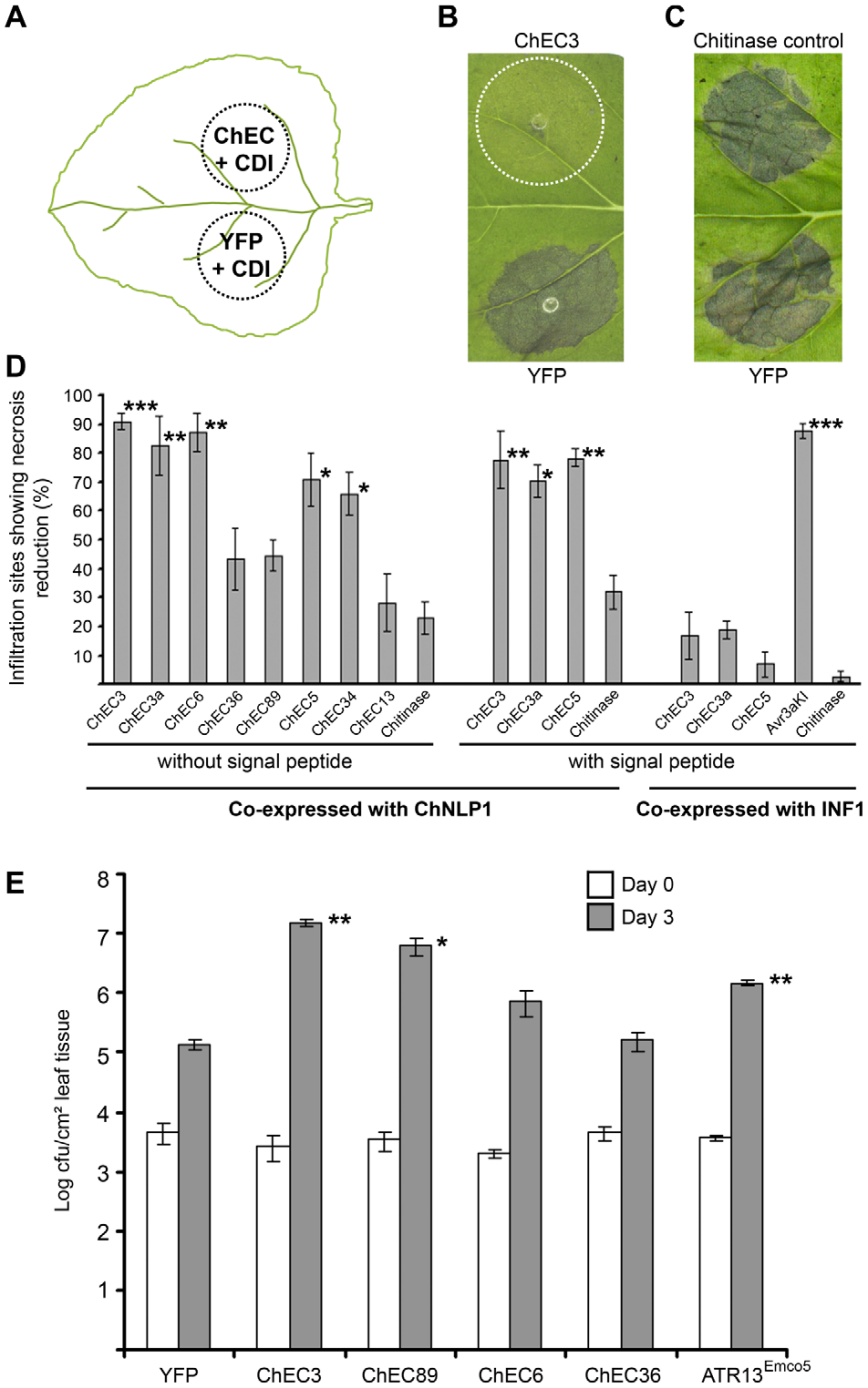
ChECs antagonizing plant cell death and supporting multiplication of plant pathogenic bacteria. (A) Infiltration scheme for the transient co-expression assay. Agrobacteria containing constructs for ChEC or YFP expression were mixed with those for cell death-inducer (CDI) expression. Mixtures were infiltrated into opposite sides of *N. benthamiana* leaves to allow pair-wise comparisons and to take account of leaf-to-leaf variation in necrosis manifestation. Thus, an infiltrated site expressing YFP/ChNLP1 was included as an internal control in every infiltrated leaf, to which the site expressing ChEC/ChNLP1 was compared. (B, C) Examples of infiltration site pairs 8 dpi. ChEC3 abolishes ChNLP1-induced necrosis (B, dotted circle), but a fungal secreted chitinase does not (C). (D) Quantification of cell death-suppressing activity of four wave 2 effectors (ChEC3, 3a, 6, 36), three wave 3 effectors (ChEC89, 34, 13) and an *in vitro*-expressed effector (ChEC5). Histograms show the proportion of sites expressing ChEC/CDI that displayed reduced necrosis compared to control sites expressing YFP/CDI. *, ** and *** indicate significant difference from the respective chitinase control with and without signal peptide at P<0,02, <0.005 and <0.0002, respectively (Student's t-test). *P. infestans* effector Avr3a^KI^ was used as positive control for suppression of INF1-induced cell death. Data represent means of at least three independent experiments, with at least 15 leaves/experiment/co-expression combination (± standard error). (E) Bacterial titers in *Arabidopsis* Col-0 leaves infected with *Pseudomonas syringae* pv *tomato* expressing ChECs as fusions with a bacterial effector mediating delivery into plant cells *via* type III secretion. *Hyaloperonospora arabidopsidis* ATR13^Emco5^ [27] and YFP were included as positive and negative controls, respectively. Colony forming units were determined 0 and 3 days after spray inoculation. * and ** indicate significant difference from the YFP control at P<0.03 and P<0.0005, respectively. Data represent means of 4 replicates (± standard error).

Targeted mutagenesis of a gene provides unambiguous genetic evidence for its contribution to fungal virulence. However, targeted replacement of pathogen effector genes frequently does not result in reproducible infection phenotypes, possibly due to functional redundancy between effectors [19], [22]. Thus, assigning virulence-related functions to ChECs remains a challenging task. However, direct expression of ChECs in plant cells allows their biological activity to be investigated in isolation from other fungal effectors. To test whether ChECs can suppress plant cell death, we transiently co-expressed them in *N. benthamiana* leaves together with cell death-inducing proteins. In brief, agrobacteria containing a vector for ChEC expression were mixed with those for cell death inducer expression and infiltrated into one half of a leaf. A control mixture, in which ChEC-carrying agrobacteria were replaced by those enabling YFP expression, was infiltrated into the other half, allowing pair-wise comparisons in the same leaf (Figure 6A). Infiltration sites co-expressing YFP and ChNLP1 showed severe confluent necrosis six to eight days after infiltration. To quantify cell death suppression activity, we determined the proportion of ChEC-expressing sites showing reduced necrosis (Figure 6B) or no reduction in necrosis (Figure 6C). We tested four wave 2 effectors and three wave 3 effectors, including ChEC3, ChEC3a and ChEC5 due to their similarity to effectors required for pathogenicity in other fungi. Co-expression of ChEC3, ChEC3a, ChEC5, ChEC6 and CHEC34 without their signal peptides reduced necrosis in 70 to 90% of the inspected infiltration site pairs (Figure 6D). In contrast, co-expression of a *C. higginsianum* chitinase without its signal peptide as negative control protein [16] resulted in significantly fewer sites showing reduced necrosis (P<0.02, Student's t-test). Western blot analysis using epitope-tagged ChNLP1 confirmed that co-expression of ChECs has no impact on ChNLP protein level *per se* (Figure S10). Thus, the observed necrosis reduction reflects ChEC activity rather than failure of ChNLP1 expression. ChEC13, ChEC36 and ChEC89 lacked statistically significant cell death-suppressing activity.

To evaluate whether the presence of the fungal signal peptide affects activity, we re-tested three of the most active suppressors of ChNLP1-induced necrosis including their signal peptides. Co-expression of ChEC3, ChEC3a or ChEC5 with their signal peptides resulted in significantly fewer sites showing reduced necrosis relative to the chitinase control with signal peptide (P<0.01). Thus, data obtained from ChEC constructs with and without signal peptide were not significantly different (P>0.3, Figure 6D). To evaluate the specificity of the cell death suppressing activity, we re-tested the same three ChECs for their ability to interfere with necrosis induced by *Phytophthora infestans* INF1, an elicitor requiring different plant signalling components [25]. ChEC3, ChEC3a or ChEC5 failed to suppress INF1-induced necrosis, whereas co-expression of Avr3a^KI^, a well-described suppressor of INF1-induced cell death [26], resulted in significant necrosis reduction in our assay (Figure 6D). Thus, ChEC3, ChEC3a and ChEC5 specifically interfere with ChNLP1-induced necrosis, but not INF1-induced necrosis.

Preliminary experiments showed that challenge of ChEC-expressing sites with the adapted tobacco pathogens *C. orbiculare* and *C. destructivum* did not result in enhanced fungal growth, possibly as a result of immune responses triggered by the agroinfiltration procedure. To further investigate the virulence function of ChECs, we tested the ability of two suppressors and two non-suppressors of ChNLP1-induced cell death to promote the multiplication of plant pathogenic bacteria. For this we used *Pseudomonas syringae* pv. *tomato* carrying the ‘effector detector vector’ to deliver ChECs *via* the bacterial type III secretion system into the cytoplasm of *Arabidopsis* cells [27]. Out of the four proteins tested, ChEC3 and CHEC89, but not ChEC6 and ChEC36, significantly enhanced bacterial virulence compared to a YFP control, presumably by suppressing host defense responses (Figure 6E).

## Discussion

The role of secreted effector proteins during infection by hemibiotrophic plant pathogens is poorly understood. The present study provides a comprehensive inventory of *in planta*-expressed effector candidates for the hemibiotrophic fungus *Colletotrichum higginsianum*. We found most biotrophy-associated ChEC genes were dramatically upregulated exclusively *in planta*, which suggests these proteins play an important role during host infection. Consecutive waves of effector gene expression were associated with key developmental transitions, indicating that distinct suites of effectors are deployed at each infection stage.

For ChECs upregulated pre-invasion (waves 1 and 2), we demonstrated focal secretion to and from appressorial penetration pores. Appressoria have been long-recognized as structures enabling turgor-driven penetration of host surface barriers [2]. We now add another level of functional complexity to this highly elaborated infection structure that has not been reported previously, namely the local release of effector proteins at a nanoscale interface formed between host and pathogen, defined by the basal penetration pore. In contrast to secreted proteins that are targeted to the inner appressorial wall where they may play structural roles [28], ChECs were specifically secreted to the penetration pore, reflecting the strong basipetal polarity associated with switch from radial (isometric) expansion to focused tip growth of the emerging penetration hypha [4]. ChEC6:mRFP and ChEC36:mRFP fusions were expressed and targeted to this pore before any ingression into the host cell had occurred. Given that *in vitro* and *in planta* appressoria are morphologically indistinguishable [29] and that these effectors were not expressed by *in vitro* appressoria, this suggests that host-derived signals, rather than developmental cues, induce ChEC expression. Moreover, these signals must be sensed before penetration hyphae emerge, presumably *via* the wall-less pore region. Unlike all other ChECs expressed as mRFP fusions, only CHEC13:mRFP was detectable during penetration of cellophane, suggesting *CHEC13* expression is developmentally linked to penetration hypha formation. Similarly, expression of the *M. oryzae* avirulence gene *ACE1*, involved in the synthesis of a secondary metabolite effector, is linked to the emergence of penetration hyphae [30].

Why are ChECs expressed and secreted at such an early stage of pathogenesis? Our ultrastructural analyses revealed host-derived cell wall material is deposited beneath appressoria before any visible penetration or structural damage to the cuticle/cell wall, indicating *Arabidopsis* perceives and responds to *C. higginsianum* appressoria before fungal entry. We propose that the fungus deploys early-expressed effectors to counteract pre-invasion host defenses and to prepare the host cell for colonization. In support of this idea, it was previously demonstrated for *C. lindemuthianum* that appressorium maturation, but not penetration, was sufficient to induce bean defense responses [31]. Similarly, *C. lindemuthianum* mutants lacking the transcription factor *Ste12* required for appressorial penetration induced a hypersensitive response and defense gene expression in non-host plants, again indicating that pathogen perception is independent of penetration [32]. *Arabidopsis* resistance to *C. higginsianum* conferred by the resistance genes *RRS1* and *RPS4* acts very early, before formation of biotrophic hyphae [33]. This suggests wave one or two effectors (or their activities) may be recognized by these resistance proteins. Shimada and co-workers [34] proposed that *C. higginsianum* is able to suppress callose wall depositions in attacked *Arabidopsis* cells. Consistent with this, we barely detected callose within the first-formed host cell wall deposits. However, these initial deposits were subsequently encrusted with a layer of β-1,3 glucan. Intriguingly, we also observed specific labelling of the host cuticle-cell wall interface directly beneath appressoria (Figure S5 G, H). Further work is required to determine whether this β-1,3 glucan is of plant or fungal origin.

The observed accumulation of wave three effectors in interfacial bodies on the surface of biotrophic hyphae is reminiscent of the *M. oryzae* biotrophic interfacial complex (BIC), in which fluorescent protein-tagged effectors also accumulate [19]. However, when biotrophic hyphae of *M. oryzae* occupy the first invaded epidermal cell, only a single BIC of approx. 1–2 µm diameter is present, whereas *C. higginsinum* hyphae are decorated with numerous interfacial bodies of ∼500 nm diameter. Khang and co-workers [35] reported that BIC-localization was correlated with effector translocation into the rice cytoplasm. However, we could not detect uptake of any ChEC:mRFP fusion protein into the host cytoplasm. Nevertheless, the ability of ChECs to enhance bacterial growth upon delivery into the plant cytoplasm and/or to suppress ChNLP1-induced cell death upon direct expression in the plant cytoplasm (i.e. without their signal peptide) raises the possibility that these effectors are translocated into host cells. It is possible that the amount of translocated ChEC:mRFP fusion protein is below the detection limits of confocal microscopy and immunogold labelling. Alternatively, the mRFP tag (28 kDa) could interfere with effector translocation, although tags as large as 50 kDa were successfully used to trace *M. oryzae* effector translocation into invaded rice cells and their neighbors [35]. The use of antibodies raised against native ChEC proteins or peptides for immunolabelling may circumvent this potential problem.

*Colletotrichum* homologs of NLPs have not been reported previously. ChNLP1 was an effective cell death inducer and was strongly and specifically upregulated at the switch from biotrophy to necrotrophy, consistent with a role in terminating the biotrophic phase of pathogenesis. The strong upregulation of *ChNLP3* and *ChNLP5* early during host penetration and biotrophy is intriguing and shows that expression of NLP-homologs is not detrimental to biotrophy *per se*. Consistent with this, the genome of the biotrophic oomycete *Hyaloperonospora arabidopsidis* contains several NLP-like genes without necrosis-inducing activity [36]. ChNLP3 and ChNLP5 lack three out of four highly conserved amino acid residues required for full NLP activity [37], and as expected, ChNLP3 did not provoke cell death in *N. benthamiana* in our assay, suggesting these proteins have adopted new functions.

We hypothesize that during initial host penetration and the intracellular biotrophic phase, *Colletotrichum* likely induces host cell damage and the release of damage-associated molecular patterns (DAMPs) and needs to secrete effectors that maintain host cell viability. In support of this, we found ChECs that suppressed cell death induced by ChNLP1, which is likely to cause disintegration of the plant plasma membrane. This suppression activity was specific for NLP1-induced cell death, since these effectors did not suppress INF1-induced necrosis. It was previously demonstrated that distinct signalling pathways mediate NLP- and INF1-induced cell death in *N. benthamiana* [25]. It is conceivable that in our co-expression assay *in planta*, *C. higginsianum* effectors interfere with ChNLP1-specific signalling components and thereby prevent amplification of a cell death signal or its spreading from cell to cell. Plant responses evoked by NLPs share some characteristics with MAMP-triggered immunity [38], [39]. The broad taxonomic distribution of NLPs in fungi, oomycetes and bacteria, and the relatively high sequence conservation of NLPs is also consistent with the classical concept of MAMPs [24]. It was suggested previously that plant cells recognize NLP action but not the protein itself and that NLP-mediated membrane disruption may release endogenous damage-associated molecular patterns (DAMPs) [37]. In view of their reciprocal expression pattern during infection, cell death-suppressing ChECs may not interfere with ChNLP1 itself or its cytolytic activity but rather with responses to DAMPs, or to other factors inducing cell death through similar pathways, that are released or triggered during biotrophic invasion. Consistent with a role in the suppression of MAMP/DAMP-triggered immunity, ChEC3 also supported the multiplication of plant pathogenic bacteria. *ChEC3* and its paralog *ChEC3a* resemble *CgDN3* from *C. gloeosporioides*, which is phylogenetically distant from *C. higginsianum* [12]. Similar to ChEC3 and ChEC3a, *CgDN3* was found to be expressed during the early biotrophic phase of *C. gloeosporioides* [14]. These authors found that a fungal mutant lacking *CgDN3* was non-pathogenic and elicited a cell death response in attacked cells, and they proposed a role for CgDN3 in interfering with plant defense. Here we provide experimental evidence that this effector family functions in host cell death suppression.

ChEC5 contains a cerato-platanin domain (CPD). CPD proteins have varied and sometimes contrasting activities, depending on the fungal pathogen and host species. This diversified protein family is prevalent in the genomes of ascomycete plant pathogens, including necrotrophs and obligate biotrophs as well as ectomycorrhizal fungi [40]–[42], suggesting an important role during plant colonization. Depending on the pathogen's lifestyle, certain members may have co-opted functions to suppress or elicit host cell death, and thus can be regarded as ‘core’ effectors deployed by many plant-associated fungi. Jeong and co-workers [17] reported the failure of appressorial penetration and early abortion of pathogenesis in *M. oryzae* mutants lacking *MSP1*, a homolog of *ChEC5*, suggesting this CPD protein is involved in establishing a biotrophic interaction with host cells. Similar to *ChEC5*, MSP1 expression was not differentially regulated *in planta* [43]. Our study provides the first evidence that a CPD protein acts as a cell death suppressor, further expanding the range of biological activities of this protein family.

Although many effectors of filamentous pathogens interfere with INF1-induced cell death and cell death resulting from effector-triggered immunity [26], [44], [45], only one effector suppressing NLP-induced cell death has been reported previously, namely *P. infestans* SNE1 [46]. Remarkably, SNE1 showed this effect when directly expressed in the plant cytosol, without its signal peptide. SNE1 carries the motif RXLX at its N-terminus, which resembles a variant of the oomycete host translocation motif RXLR, and was shown to mediate effector uptake [47], [48]. Similarly, ChEC3, ChEC3a, ChEC5, ChEC6 and CHEC34 also exert cell death-suppressing activity without their signal peptides. Despite the lack of any RXLR-like or other shared amino acid motif in these proteins, this finding suggests they act intracellularly after translocation into host cells. Thus, the necrosis-suppressing effect of full-length ChECs could result from their re-entry after being secreted by the plant cell.

In conclusion, the extreme stage-specificity and reciprocal expression patterns of cell death inducers and suppressors raise the possibility that *Colletotrichum* utilizes the same type of programmed cell death at the onset of necrotrophic growth that it must previously suppress during biotrophy. This would imply that effector-targeted components of the signalling cascade required for NLP-induced cell death become compatibility factors for the later necrotrophic stage of infection. In addition, the focal release of effectors is revealed as a new function for the appressoria of plant pathogenic fungi. The small contact zone delimited by the appressorial penetration pore can be regarded as a highly localized battleground to which both opponents target their weapons, even before the outer host barriers are breached. An intriguing finding was the tight regulation and plant-responsiveness of most effector genes. Future goals will be to decipher the nature of the plant signal(s) inducing effector gene expression and how they are sensed by the pathogen. A better understanding of host perception by phytopathogenic fungi is likely to provide novel strategies for the control of many economically important crop diseases through chemical intervention or plant breeding.

## Materials and Methods

### Fungal and plant material

*C. higginsianum* isolate IMI349063A was used for EST generation and as background strain for fungal transformations. The abaxial surface of detached *Arabidopsis* leaves was inoculated and incubated as described previously [16]. Epidermal peels were prepared by adhering the adaxial surface with double-sided tape and quickly removing the epidermis using tweezers. Pieces of remaining mesophyll were excised quickly and the peels (∼15 mm^2^ per leaf) were flash-frozen in liquid nitrogen. The following infection stages were sampled using this technique: 5 hpi (germlings; RT-PCR), 22 hpi (unpenetrated appressoria *in planta*; RT-PCR, qRT-PCR and EST generation), 40 hpi (penetrated appressoria *in planta* with nascent biotrophic hyphae; RT-PCR, qRT-PCR and EST generation) and mock-inoculated leaves (RT-PCR and EST generation). Microscopic spot-checks of infected material ensured the absence of biotrophic hyphae in samples representing unpenetrated appressoria *in planta* and the absence of necrotrophic hyphae in samples representing penetrated appressoria with nascent biotrophic hyphae. Sectors including the first appearing pin-point water-soaked lesions (∼60 hpi) were sampled for the switch between biotrophy and necrotrophy (Figure S3). Macerated leaves at 72 hpi represented late necrotrophy. Saprotrophic mycelium and conidia were produced as described previously [13]. *In vitro* appressoria and germlings formed on an unpenetratable surface were obtained by incubating spores on polystyrene [29]. Formation of ‘pseudo biotrophic hyphae’ within cellophane membranes was achieved using autoclaved dialysis tubing (Visking, Roth).

### Bioinformatic screening for ChECs

Details about library preparation, sequencing techniques, strategies to maximize gene discovery as well as EST assembly statistics can be found in Text S1. ORFs were predicted from EST contigs with the *Fusarium* matrix of BESTORF (Molquest package, Softberry). Solubly secreted proteins were identified following published guidelines [49]. Secreted proteins (and their corresponding contigs) for which no significant (<1e-5) BLASTX, BLASTN or TBLASTX [50] match could be obtained in GenBank's protein, nucleotide and EST databases, respectively, were defined as ChECs, supplemented with proteins resembling previously described fungal effectors. TBLASTN identified orthologs in the *C. graminicola* genome (http://www.broadinstitute.org/annotation/fungi/). ChEC-encoding contigs depleted in ESTs from the late necrotrophic phase (<15%) were defined as ‘biotrophy-associated’. The inventory of biotrophy-associated ChECs was manually curated to remove (i) incomplete ORFs with missing C-termini present in the genome showing homology to known proteins (ii) ORFs <20 residues and (iii) artifactual ORFs with monotonous sequences. For further analysis, we prioritized ChECs that resembled previously identified effectors and/or displayed high expression levels in biotrophy-relevant stages as determined by their EST read counts in our non-normalized libraries (see Text S1 for details on library preparation)

### Quantitative real-time PCR

Three biological replicates were obtained for each sampled fungal stage. cDNA was synthesized from 1 µg total RNA using the iScript cDNA synthesis kit (Bio-Rad) in a volume of 20 µL. Two µL of cDNA (5 ng/µL) were amplified in 1X iQ SYBR Green Supermix (Bio-Rad) with 1.6 µM primers using the iQ5 Real-time PCR detection system (Bio-Rad). Specific primers (see Text S1) amplified fragments ranging from 106 to 329 bp with efficiencies ranging from 97 and 123%. GeNorm (http://medgen.ugent.be/wjvdesomp/genorm/) was used to assess expression stability of five commonly used reference genes of which α-tubulin and actin were most stable (stability value 0.047 and 0.051, respectively) and used to normalize gene expression [51].

### Localization of ChECs, fungal transformation and functional assays

To localize ChECs by fluorescent protein-tagging, genes including at least 1.5 kb or the entire upstream intergenic region were amplified from DNA, followed by TOPO cloning (Invitrogen), sequence verification and shuttling into pFPL-R, a binary destination vector providing C-terminal translational fusions to mRFP. This vector was created and kindly provided by Dr. M. Farman (Univ. of Kentucky, Lexington, KY). Fungal transformation was carried out as described by Huser and co-workers [52]. A detailed description of the cloning and the agroinfiltration procedure used for transient expression in *N. benthamiana* is described in Text S1. The ‘effector detector vector’-based bacterial multiplication assay was performed according to Sohn *et al.* [27].

### Light microscopy

For cellulose acetate-stripping, 5% (w/v) cellulose acetate (Sigma-Aldrich) in acetone was brushed on the inoculated leaf. After complete acetone evaporation, the cellulose acetate coating was stripped off. Confocal images were obtained with Leica TCS SP2 or Zeiss LSM 700 confocal scanning microscopes. Excitation for imaging GFP fluorescence used the 488 nm laser line and emission was detected at 492–550 nm. For imaging mRFP, excitation was at 563 (Leica) or 555 nm (Zeiss) and emission was detected at 566–620 (Leica) or 557–600 nm (Zeiss). To discriminate mRFP emission from autofluorescence, we used spectral imaging in the lambda mode of a Zeiss LSM 510 microscope. Using the Meta detector and 545 nm excitation line, image stacks with 558–648 nm emission were recorded. To separate mixed fluorescent signals and resolve the spatial distribution of mRFP fluorescence, linear unmixing was employed using the mRFP emission spectrum and several autofluorescence spectra as references.

### Electron microscopy

Cellulose acetate replicas and the stripped leaf surface were imaged with a Zeiss Supra 40VP scanning electron microscope at 10 kV. Stripped leaf surfaces were fixed using a cryo-preparation device (Emitech Technologies, Ringmer). Specimens were frozen in liquid nitrogen slush and sputter-coated with palladium after sublimation of surface ice (Polaron Sputter Coater SC 7600, Quorum Technologies). Samples for ultrastructural observation were processed according to [13]. For immunogold labelling, infected plant material was fixed in 4% (w/v) *p*-formaldehyde and 0.5% (v/v) glutaraldehyde in 0.05 M sodium cacodylate buffer, pH 6.9, for 2 h. After progressive low-temperature dehydration in a graded water-ethanol series [53], samples were embedded in LR White resin (Plano GmbH, Wetzlar, Germany). For immunogold labelling, we used procedures described previously [54]. Rabbit polyclonal anti-mRFP antibody (R10367, Molecular Probes) and mouse monoclonal anti-ß-1,3-glucan antibody (Biosupplies Australia Pty., Parkville, Australia) were both applied at dilutions of 1 in 500. Goat anti-rabbit and goat anti-mouse IgG antibodies conjugated with 5 or 10 nm colloidal gold particles (British Biocell International, Cardiff, UK) were used as secondary antibodies.

### Accession numbers

ChEC3 (HE651156), ChEC3a (HE651158), ChEC4 (HE651159), ChEC5 (HE651160), ChEC6 (HE651161), ChEC7 (HE651162), ChEC9 (HE651164), ChEC13 (HE651168), ChEC34 (HE651193), ChEC36 (HE651195), ChEC51 (HE651213), ChEC56 (HE651219), ChEC88 (HE651251), ChEC89 (HE651252), ChToxB (HE651256), ChNLP1 (HE651257), ChNLP3 (HE651259), ChNLP5 (HE651261). Accession numbers for the entirety of ChECs can be retrieved from Table S1. ESTs were submitted to the EBI Sequence Read Archive under the accession number ERP001241 (http://www.ebi.ac.uk/ena/data/view/ERP001241).

## Supporting Information

Figure S1 **Sequence diversification of selected ChECs.** (A) Southern blot analysis using genomic DNA of 21 different *Colletotrichum* species or isolates. Hybridization conditions allowed 25% nucleotide mismatches. Note the low sequence conservation or absence of *ChEC* genes outside the *C. destructivum* species aggregate. Only *ChEC2* was detectable outside the *C. destructivum* species aggregate (asterisk) and was confirmed by BLAST to have 76% identical base pairs with the *C. graminicola* homolog, consistent with the hybridization conditions used. *ChEC3* is only detectable in *C. higginsianum* strains, isolated from *Matthiola incana* (a), *Raphanus sativus* (b) and *Brassica spp.* (c). A control gene encoding a non-secreted calpain protease, although absent from most sequenced ascomycete genomes [29], is conserved in all species tested. The ethidium bromide-stained agarose gel before blotting is shown below as loading control. From left to right, the following species and isolates were analyzed: *C. higginsianum* IMI349063A (reference strain), *C. capsici* LARS 141, *Glomerella magna* LARS 688, *C. malvarum* LARS 629, *C. gloeosporioides* LARS 074, *C. trifolii* LARS 972, *C. lagenarium* 104-T, *C. gloeosporioides* LARS 224, *C. graminicola* M1.001, *C. truncatum* LARS 060, *C. higginsianum* Ch90-M3, CH93-M1, AR 3-1, NBRC6182, Abo 1-1, Abp 3-1 and MAFF 305968, *C. destructivum* N150, LARS 056 and LARS 709, *C. linicola* IMI 103844. (B) Alignment of ChEC3, ChEC3a and *C. gloeosporioides* CgDN3 protein sequences (above) and corresponding secondary structure predictions of the mature proteins (below). Amino acid residues identical in all three proteins are indicated in red, those identical in ChEC3 and ChEC3a are shaded in grey. The predicted signal peptide cleavage site is marked with a triangle. The green arrow indicates the conserved position of a phase 2-intron, which splits the codon for the conserved histidine residue between the second and third base. A black arrow indicates the only single nucleotide polymorphism identified by sequencing *ChEC3* and *ChEC3a* genes from 17 different *C. higginsianum* isolates, resulting in an exchange of the aspartate with asparagine in the protein sequence of ChEC3a in *C. higginsianum* isolate CH93-M1 C, coil; E, strand; H, helix.
(TIF)

Figure S2 **ChEC4 and ChEC9 carry functional nuclear localization signals (NLS).** (A, B) Amino acid sequences of ChEC4 (A) and ChEC9 (B). The predicted signal peptides and NLS are in bold face and underlined, respectively. The coloured letters in the ChEC4 sequence indicate three nearly identical tandem amino acid repeats which form modules encompassing the predicted bipartite NLS. (C, D) Transient co-expression of mCherry and C-terminally GFP-tagged ChEC4 or ChEC9 in *N. benthamiana*. Expression of ChEC4-GFP (C) and ChEC9-GFP (D) without their predicted signal peptides results in strong accumulation in plant nuclei. In contrast, the similar-sized mCherry is equally distributed in cyto- and nucleoplasm.
(TIF)

Figure S3 **Illustration of infected leaf samples representing the transition between biotrophy and necrotrophy, and late necrotrophy.** Leaves were densely inoculated (see methods) and incubated until onset of symptom development (top leaf, 60 hpi) or complete maceration (bottom leaf, 72 hpi). Symptoms were photographed on a light box and samples for microscopy were stained with Trypan blue as described by Takahara *et al.* (2009). To isolate RNA from biotrophic hyphae switching to necrotrophy, sectors (dotted line) surrounding the first pin-point water-soaked lesions (arrow) were harvested. Within these lesions, thin necrotrophic hyphae proliferate extensively (A), similar to the fungal growth within completely macerated leaves at the late necrotrophic stage (B). However, in the area surrounding these pin-point lesions, most infections comprised biotrophic hyphae undergoing the switch to necrotrophy (C), as indicated by the emergence of nascent necrotrophic hyphae (arrowheads). Scale bars: 5 mm (leaves) or 20 µm (microscope images). BH, previously biotrophic hyphae. NH, necrotrophic hyphae.
(TIF)

Figure S4 **Transmission electron microscopy immunogold detection of ChEC36:mRFP using antibodies to mRFP.** (A) Labelled protein inclusion bodies within fungal vacuoles (FV). (B) Unlabelled biotrophic hyphae (BH). PV, plant vacuole. (C) Labelled appressorial pore (P) surrounded by an unlabelled pore wall overlay (arrowheads). (D) Tangential section through a pore wall overlay (black asterisks) labelled on the inner surface. (E) Pore labelling external to the appressorial plasma membrane. The location of the plasma membrane between the appressorial cytoplasm (AC) and the pore wall overlay (black asterisk) is indicated with arrows. (F) Wild-type appressorium showing absence of any labelling. White asterisks, appressorial cell wall. P, penetration pore. PW, plant cell wall. WD, plant cell wall deposits. Scale bars, 500 nm.
(TIF)

Figure S5 **Immunodetection of β-1,3-glucans in the appressorial pore wall overlay and in host cells at appressorial attack sites.** Immunofluorescence (A–F) and immunogold labelling (G, H) with antibodies recognizing β-1,3-glucan (10 nm colloidal gold) on wild-type (A–G) and transformant appressoria expressing ChEC36:RFP. (A–F) Appressoria detached from the leaf surface by cellulose acetate-stripping (compare Figure 3M) and labelled by floating on antibody solutions. Cells are viewed from the plant-exposed underside using bright-field (A, D), confocal laser scanning microscopy (B, E) and overlay of bright-field and fluorescence channels (C, F). Antibodies have entered the cells through the basal appressorial penetration pore (white arrows) to label the pore wall overlay, which forms a ring around the pore. (G, H) Cross-sections through the base of appressoria, close to the penetration pore. β-1,3-glucan is detected in the pore wall overlay (arrows) and at the interface between the plant cuticle (arrowheads) and the plant cell wall (PW). (H) β-1,3-glucan is not detected in a pad of host wall deposits (WD) beneath an appressorium but is present in a layer (asterisks) outside the pad. Section was double-labelled with antibodies to mRFP (5 nm colloidal gold). AW, appressorial cell wall. AC, appressorial cytoplasm. Scale bars, 500 nm (G, H), 5 µm (A–C), 2 µm (D–F).
(TIF)

Figure S6 **Confocal laser scanning microscopy reveals ChEC delivery to the intimate biotrophic interface between host and pathogen.** (A, B) Transformant biotrophic hypha expressing the wave 3 effector ChEC89:GFP. (C) Maximum fluorescence intensity overlay of a transformant biotrophic hypha expressing ChEC89:mRFP. Note the plant cell wall is labelled (arrowhead). White arrows in B and C indicate fluorescent foci. (D, E, F) Transformant biotrophic hypha expressing ChEC13:mRFP (wave 3 effector). The asterisk in F indicates the location of the appressorium. (G, H, I) Transformant biotrophic hypha expressing ChEC3:mRFP (wave 2 effector). Note the fluorescence-depleted hyphal tips (arrowheads in H). (I) Epidermal cell with intracellular biotrophic hypha and weak fluorescence in the apoplastic space (*) enlarged by plasmolysis. Arrowheads demarcate the host plasma membrane. (A, D, G) Bright field images. (B, E, F) Maximum fluorescence intensity projections. (C, H, I) Maximum fluorescence intensity overlays. A, appressorium. BH, biotrophic hypha. V, vacuole of the host protoplast. Scale bars: 5 µm (A, D), 10 µm (C, F, G), 20 µm (I).
(TIF)

Figure S7 **Transformant biotrophic hyphae expressing CHEC89:mRFP viewed with confocal laser scanning microscopy.** Epidermal cell infected by a biotrophic hypha (arrows) showing fluorescence in the apoplastic space (*) enlarged by plasmolysis. Arrowheads demarcate the host plasma membrane. V, vacuole of the host protoplast. Scale bar: 10 µm. See also Fig. 4E. (A) identical to Fig. 4E. (B) corresponding brightfield image.
(TIF)

Figure S8 **Most ChEC:mRFP fusion proteins are plant-induced and not detectable during penetration of an artificial substratum.** Transformants expressing the wave 2 effectors ChEC36:mRFP (A, B) and ChEC6:mRFP (C, D; focus on appressorial penetration pores (arrows) or the wave 3 effectors ChEC89:mRFP (E, F), ChEC34:mRFP (G, H) or ChEC13:mRFP (I, J) were inoculated onto cellophane membranes. (A, C, E, G, I) Bright field images. (B, D, F, H, J) Maximum fluorescence intensity projections. Scale bars: 10 µm (C, E) and 5 µm (A, G, I). A, appressorium. C, conidia. H, pseudo biotrophic hyphae growing inside cellophane.
(TIF)

Figure S9 **Transmission electron microscopy immunogold detection of ChEC34:mRFP (wave 3 effector).** (A) Interfacial bodies of transformant biotrophic hyphae expressing ChEC34:mRFP are labelled. (B) Interfacial bodies of wild-type biotrophic hyphae are unlabelled. Black arrows, interfacial bodies. Scale bar: 500 nm.
(TIF)

Figure S10 **ChNLP1 expression levels are not affected by co-expression of ChECs.** ChNLP1 was cloned into a plant expression vector providing a C-terminal translational fusion with a hemagglutinin (HA) tag (ChNLP1-HA). Similar to untagged ChNLP1, ChNLP1-HA was able to induce necrosis, which was found to be suppressable upon ChEC co-expression. Before onset of visible necrotic symptoms (three days after infiltration), eight leaf discs from different sites expressing ChEC/cell death inducer were pooled, likewise their corresponding sites expressing YFP/cell death inducer. ChNLP1-HA protein levels in ChEC3/ChNLP1-HA or ChEC5/ChNLP1-HA pools were compared to those of the corresponding YFP/ChNLP1-HA pools by Western blot analysis. ChEC3 and ChEC5 were expressed either with (+SP) or without (−SP) their signal peptides. Using an anti-HA antibody, full-length ChNLP1-HA (30 kDa expected molecular mass) could be detected, as well as three additional bands between 25 and 30 kDa, indicating partial protein cleavage had occurred. There was no major difference in band intensities between ChEC- and YFP-expressing infiltration site pairs, suggesting that co-expression of ChECs, with or without signal peptide, has no impact on ChNLP protein level *per se.* PS, Ponceau red stain.
(TIF)

Table S1 **Inventory of biotrophy-associated Colletotrichum higginsianum effector candidates (ChECs).** For any ChEC identified, the table lists (a) ENA accession numbers, (b) identifiers refering to the *C. higginsianum* genome annotated by the Broad institute, (c) protein length, (d) number of cysteines, (e) numbers of homolgues in *C. graminicola* and (f) predicted motifs or homologies to known genes.
(DOC)

Table S2 **Redundancy of ESTs from plant-penetrating appressoria as a measure for gene expression level: a survey of the top 30 contigs containing the highest numbers of ESTs from plant-penetrating appressoria.** The table lists (a) number of ESTs per contig, (b) information on the nearest informative homologue given by BLAST, (c) the presence of a signal peptide, (d) ChEC IDs.
(DOC)

Text S1 **Supporting materials and methods.** Details about (a) employed sequencing and normalization techniques, including assembly statistics, (b) RNA isolation and library preparation, (c) protein domain and motif searches, (d) Sequencing *ChEC3* and *ChEC3a* alleles, (e) RT-PCR, (f) transient expression in *N. benthamiana*, (g) Western and Southern blot analyses and (h) Cloning procedures and primers used.
(DOCX)
