# Correction: Sequential Delivery of Host-Induced Virulence Effectors by Appressoria and Intracellular Hyphae of the Phytopathogen *Colletotrichum higginsianum*

**DOI:** 10.1371/annotation/0f398a0c-dfda-4277-b172-4ff9cb31aec3

**Published:** 2012-08-30

**Authors:** Jochen Kleemann, Linda J. Rincon-Rivera, Hiroyuki Takahara, Ulla Neumann, Emiel Ver Loren van Themaat, H. Charlotte van der Does, Stéphane Hacquard, Kurt Stüber, Isa Will, Wolfgang Schmalenbach, Elmon Schmelzer, Richard J. O'Connell

The fifth author's name was abbreviated incorrectly in the citation. The correct citation is: Kleemann J, Rincon-Rivera LJ, Takahara H, Neumann U, Ver Loren van Themaat E, et al. (2012) Sequential Delivery of Host-Induced Virulence Effectors by Appressoria and Intracellular Hyphae of the Phytopathogen Colletotrichum higginsianum. PLoS Pathog 8(4): e1002643. doi:10.1371/journal.ppat.1002643

