# Supplementary figures and images for: Sequential Delivery of Host-Induced Virulence Effectors by Appressoria and Intracellular Hyphae of the Phytopathogen Colletotrichum higginsianum
Source: PLoS Pathog. 2012 Apr 5;8(4):e1002643. doi: 10.1371/journal.ppat.1002643 (PMC3320591; doi:10.1371/journal.ppat.1002643)

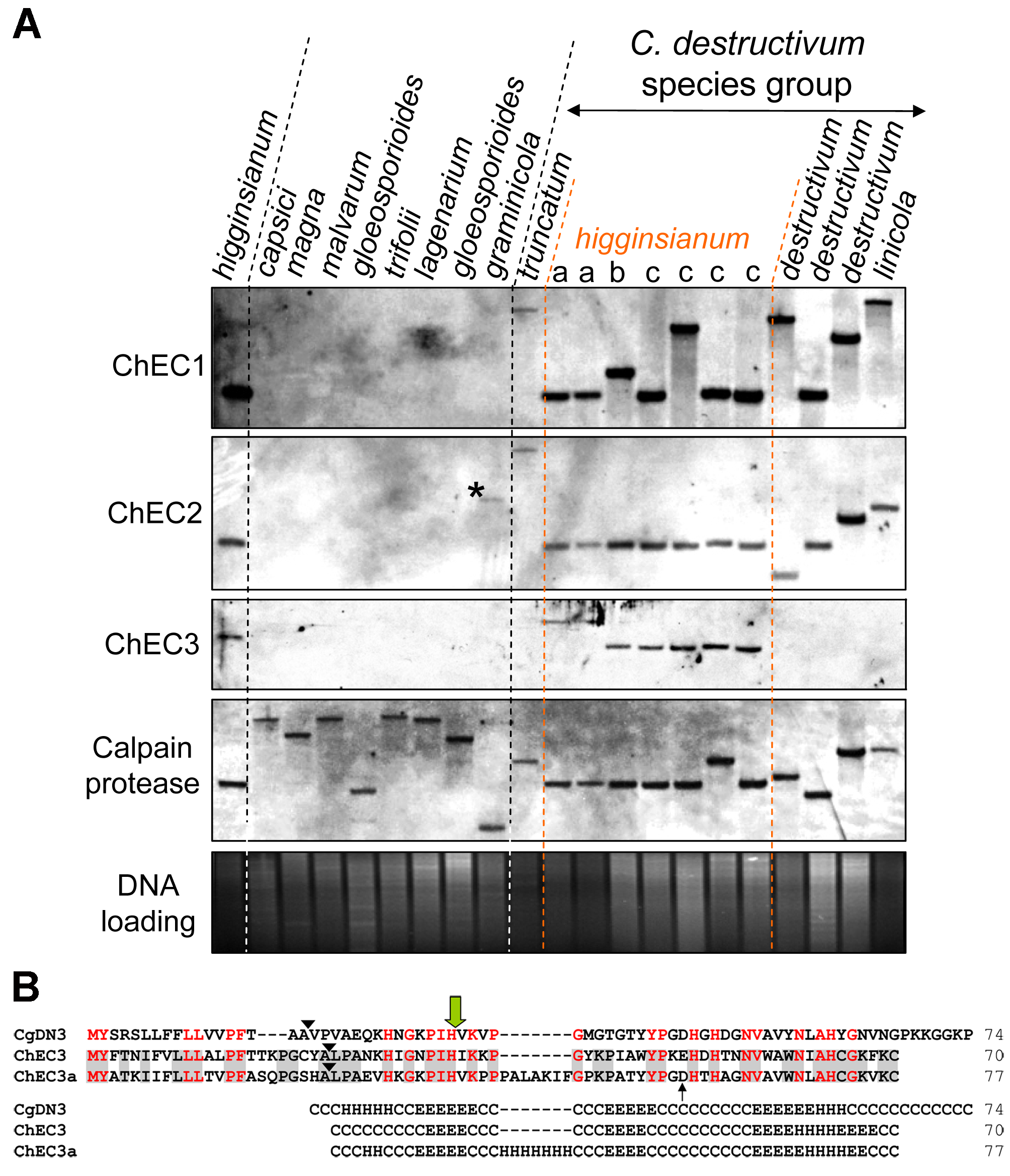

Supplement: Figure S1 — Sequence diversification of selected ChECs. (A) Southern blot analysis using genomic DNA of 21 different Colletotrichum species or isolates. Hybridization conditions allowed 25% nucleotide mismatches. Note the low sequence conservation or absence of ChEC genes outside the C. destructivum species aggregate. Only ChEC2 was detectable outside the C. destructivum species aggregate (asterisk) and was confirmed by BLAST to have 76% identical base pairs with the C. graminicola homolog, consistent with the hybridization conditions used. ChEC3 is only detectable in C. higginsianum strains, isolated from Matthiola incana (a), Raphanus sativus (b) and Brassica spp. (c). A control gene encoding a non-secreted calpain protease, although absent from most sequenced ascomycete genomes [29], is conserved in all species tested. The ethidium bromide-stained agarose gel before blotting is shown below as loading control. From left to right, the following species and isolates were analyzed: C. higginsianum IMI349063A (reference strain), C. capsici LARS 141, Glomerella magna LARS 688, C. malvarum LARS 629, C. gloeosporioides LARS 074, C. trifolii LARS 972, C. lagenarium 104-T, C. gloeosporioides LARS 224, C. graminicola M1.001, C. truncatum LARS 060, C. higginsianum Ch90-M3, CH93-M1, AR 3-1, NBRC6182, Abo 1-1, Abp 3-1 and MAFF 305968, C. destructivum N150, LARS 056 and LARS 709, C. linicola IMI 103844. (B) Alignment of ChEC3, ChEC3a and C. gloeosporioides CgDN3 protein sequences (above) and corresponding secondary structure predictions of the mature proteins (below). Amino acid residues identical in all three proteins are indicated in red, those identical in ChEC3 and ChEC3a are shaded in grey. The predicted signal peptide cleavage site is marked with a triangle. The green arrow indicates the conserved position of a phase 2-intron, which splits the codon for the conserved histidine residue between the second and third base. A black arrow indicates the only single nucleotide polymorphism i [file ppat.1002643.s001.tif]

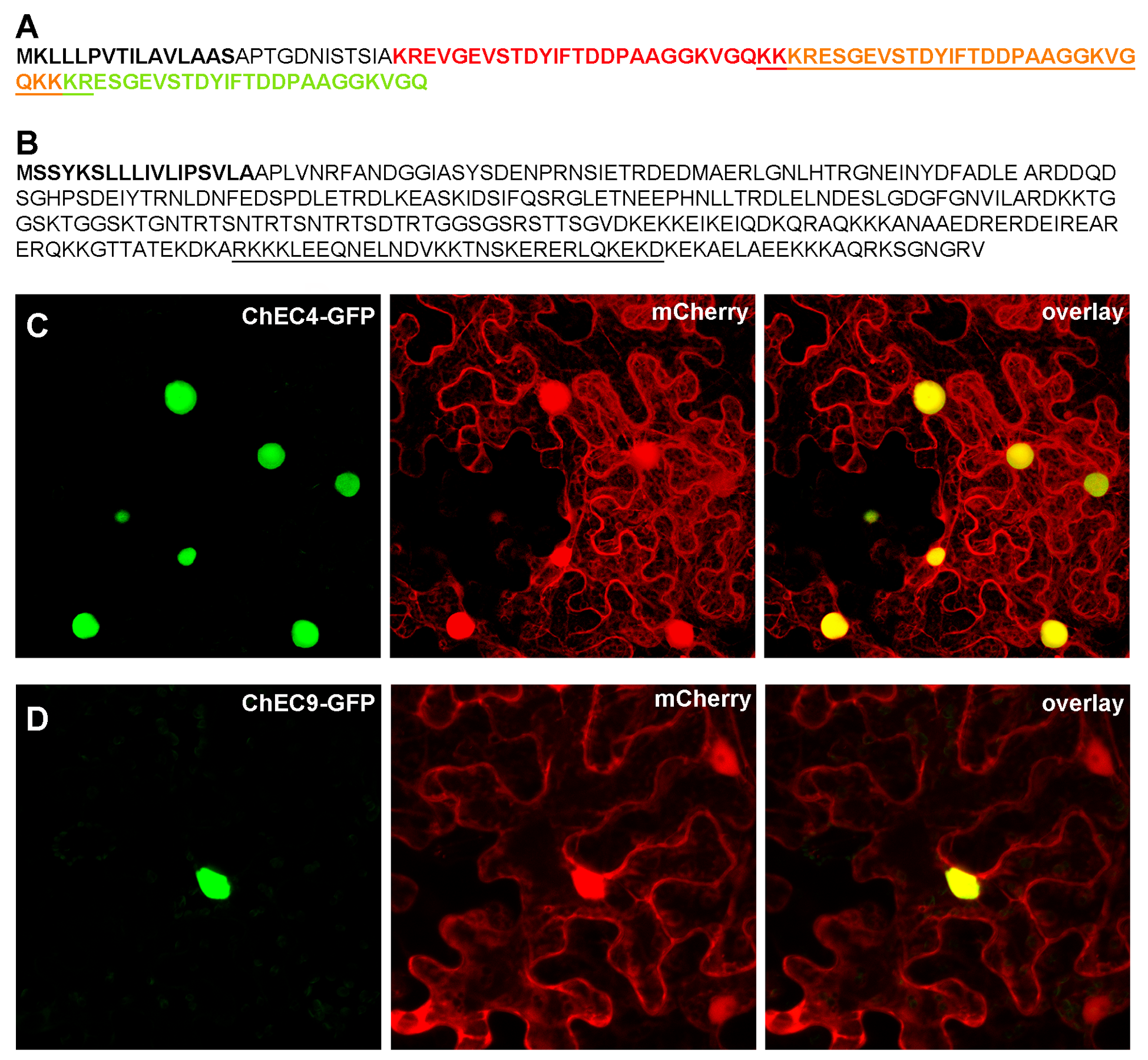

Supplement: Figure S2 — ChEC4 and ChEC9 carry functional nuclear localization signals (NLS). (A, B) Amino acid sequences of ChEC4 (A) and ChEC9 (B). The predicted signal peptides and NLS are in bold face and underlined, respectively. The coloured letters in the ChEC4 sequence indicate three nearly identical tandem amino acid repeats which form modules encompassing the predicted bipartite NLS. (C, D) Transient co-expression of mCherry and C-terminally GFP-tagged ChEC4 or ChEC9 in N. benthamiana. Expression of ChEC4-GFP (C) and ChEC9-GFP (D) without their predicted signal peptides results in strong accumulation in plant nuclei. In contrast, the similar-sized mCherry is equally distributed in cyto- and nucleoplasm. (TIF) [file ppat.1002643.s002.tif]

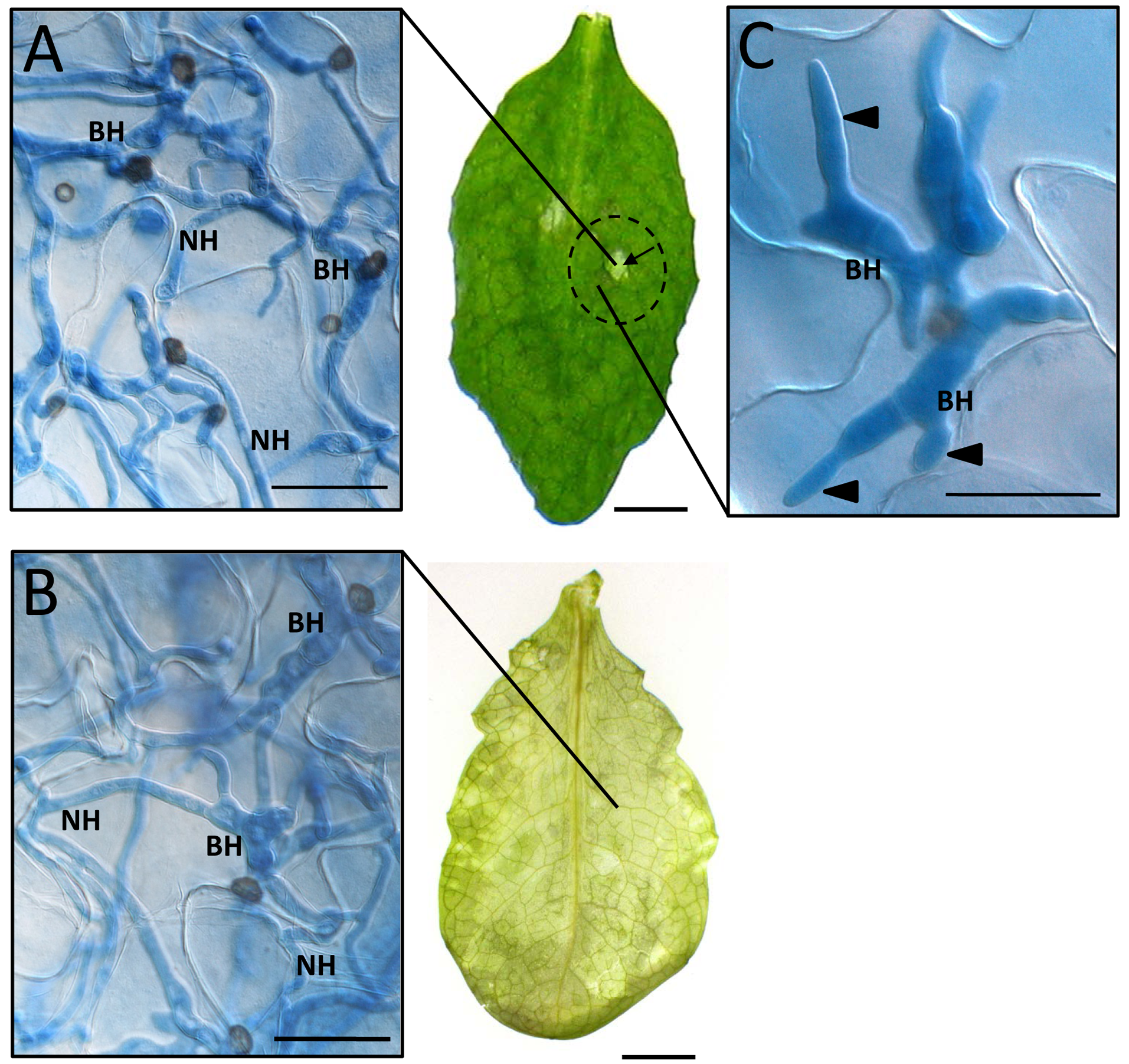

Supplement: Figure S3 — Illustration of infected leaf samples representing the transition between biotrophy and necrotrophy, and late necrotrophy. Leaves were densely inoculated (see methods) and incubated until onset of symptom development (top leaf, 60 hpi) or complete maceration (bottom leaf, 72 hpi). Symptoms were photographed on a light box and samples for microscopy were stained with Trypan blue as described by Takahara et al. (2009). To isolate RNA from biotrophic hyphae switching to necrotrophy, sectors (dotted line) surrounding the first pin-point water-soaked lesions (arrow) were harvested. Within these lesions, thin necrotrophic hyphae proliferate extensively (A), similar to the fungal growth within completely macerated leaves at the late necrotrophic stage (B). However, in the area surrounding these pin-point lesions, most infections comprised biotrophic hyphae undergoing the switch to necrotrophy (C), as indicated by the emergence of nascent necrotrophic hyphae (arrowheads). Scale bars: 5 mm (leaves) or 20 µm (microscope images). BH, previously biotrophic hyphae. NH, necrotrophic hyphae. (TIF) [file ppat.1002643.s003.tif]

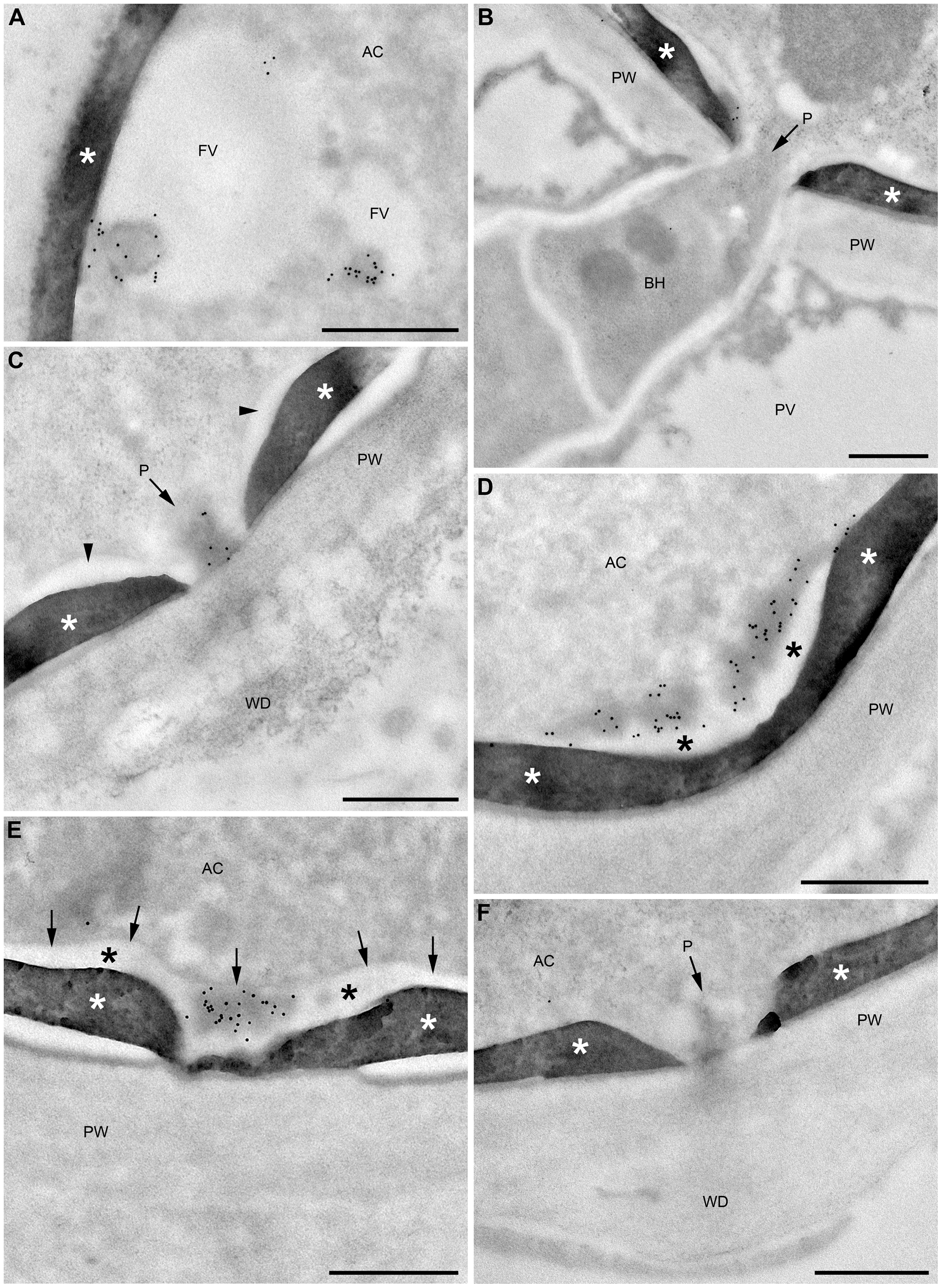

Supplement: Figure S4 — Transmission electron microscopy immunogold detection of ChEC36:mRFP using antibodies to mRFP. (A) Labelled protein inclusion bodies within fungal vacuoles (FV). (B) Unlabelled biotrophic hyphae (BH). PV, plant vacuole. (C) Labelled appressorial pore (P) surrounded by an unlabelled pore wall overlay (arrowheads). (D) Tangential section through a pore wall overlay (black asterisks) labelled on the inner surface. (E) Pore labelling external to the appressorial plasma membrane. The location of the plasma membrane between the appressorial cytoplasm (AC) and the pore wall overlay (black asterisk) is indicated with arrows. (F) Wild-type appressorium showing absence of any labelling. White asterisks, appressorial cell wall. P, penetration pore. PW, plant cell wall. WD, plant cell wall deposits. Scale bars, 500 nm. (TIF) [file ppat.1002643.s004.tif]

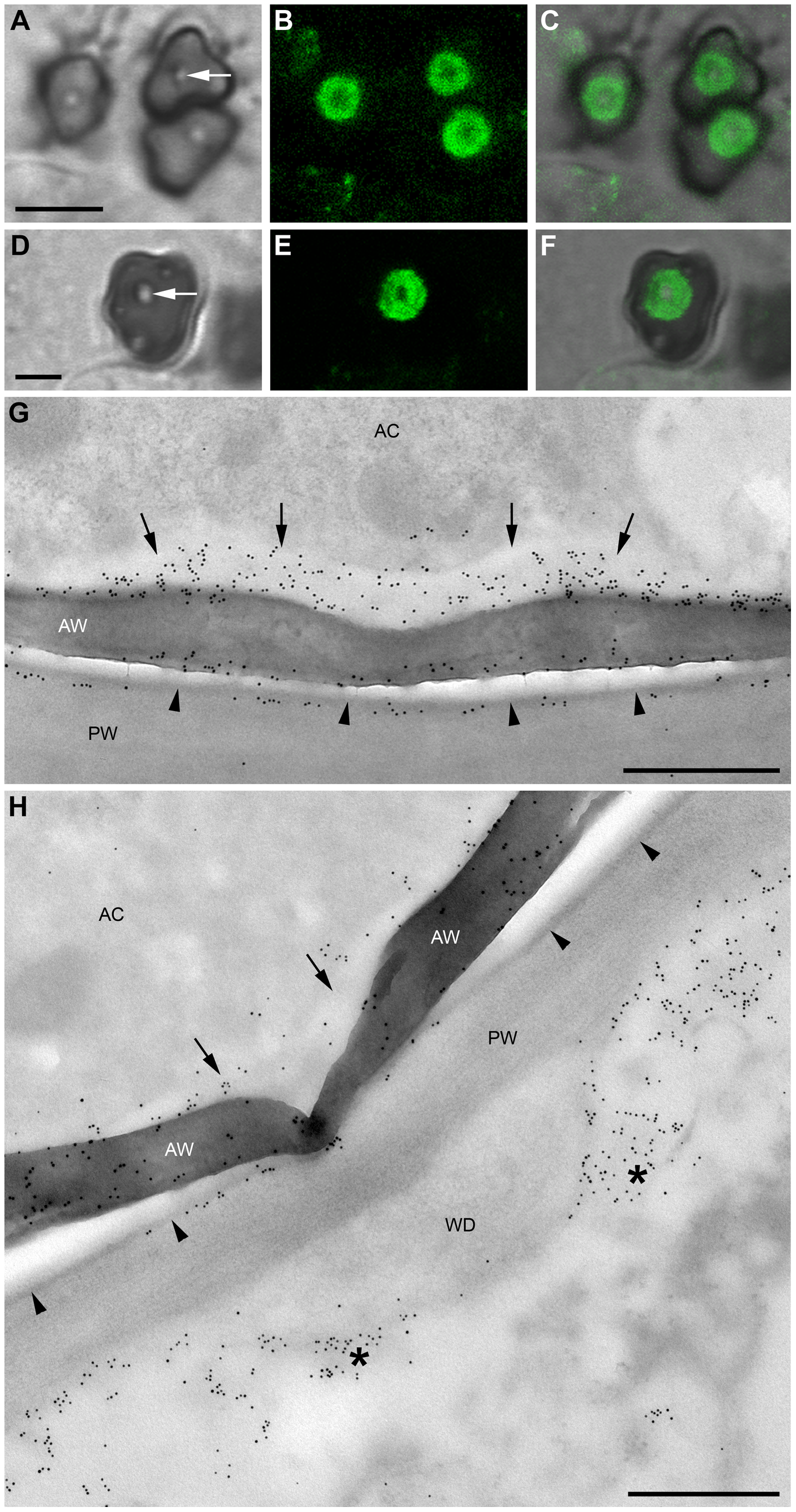

Supplement: Figure S5 — Immunodetection of β-1,3-glucans in the appressorial pore wall overlay and in host cells at appressorial attack sites. Immunofluorescence (A–F) and immunogold labelling (G, H) with antibodies recognizing β-1,3-glucan (10 nm colloidal gold) on wild-type (A–G) and transformant appressoria expressing ChEC36:RFP. (A–F) Appressoria detached from the leaf surface by cellulose acetate-stripping (compare Figure 3M) and labelled by floating on antibody solutions. Cells are viewed from the plant-exposed underside using bright-field (A, D), confocal laser scanning microscopy (B, E) and overlay of bright-field and fluorescence channels (C, F). Antibodies have entered the cells through the basal appressorial penetration pore (white arrows) to label the pore wall overlay, which forms a ring around the pore. (G, H) Cross-sections through the base of appressoria, close to the penetration pore. β-1,3-glucan is detected in the pore wall overlay (arrows) and at the interface between the plant cuticle (arrowheads) and the plant cell wall (PW). (H) β-1,3-glucan is not detected in a pad of host wall deposits (WD) beneath an appressorium but is present in a layer (asterisks) outside the pad. Section was double-labelled with antibodies to mRFP (5 nm colloidal gold). AW, appressorial cell wall. AC, appressorial cytoplasm. Scale bars, 500 nm (G, H), 5 µm (A–C), 2 µm (D–F). (TIF) [file ppat.1002643.s005.tif]

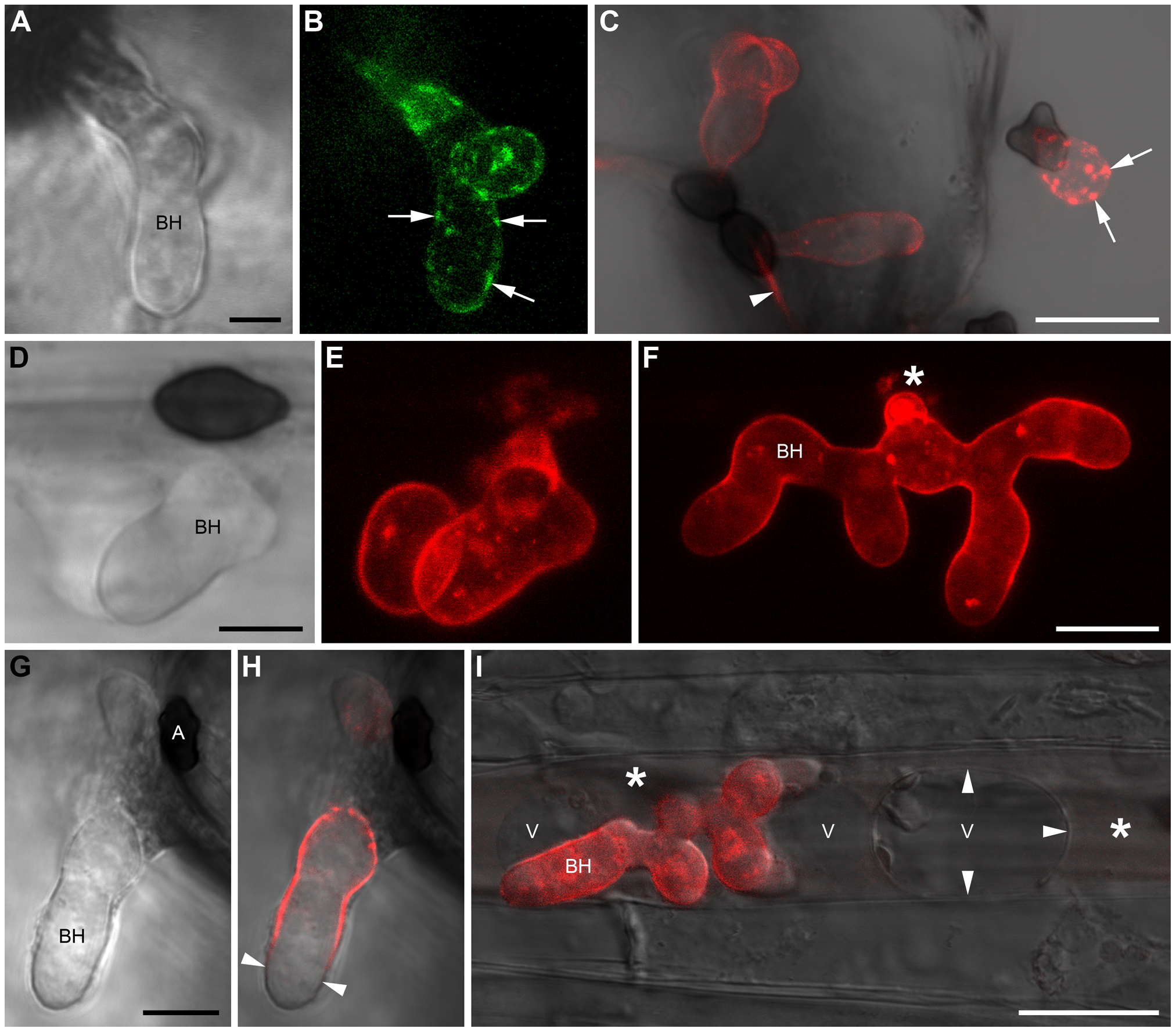

Supplement: Figure S6 — Confocal laser scanning microscopy reveals ChEC delivery to the intimate biotrophic interface between host and pathogen. (A, B) Transformant biotrophic hypha expressing the wave 3 effector ChEC89:GFP. (C) Maximum fluorescence intensity overlay of a transformant biotrophic hypha expressing ChEC89:mRFP. Note the plant cell wall is labelled (arrowhead). White arrows in B and C indicate fluorescent foci. (D, E, F) Transformant biotrophic hypha expressing ChEC13:mRFP (wave 3 effector). The asterisk in F indicates the location of the appressorium. (G, H, I) Transformant biotrophic hypha expressing ChEC3:mRFP (wave 2 effector). Note the fluorescence-depleted hyphal tips (arrowheads in H). (I) Epidermal cell with intracellular biotrophic hypha and weak fluorescence in the apoplastic space (*) enlarged by plasmolysis. Arrowheads demarcate the host plasma membrane. (A, D, G) Bright field images. (B, E, F) Maximum fluorescence intensity projections. (C, H, I) Maximum fluorescence intensity overlays. A, appressorium. BH, biotrophic hypha. V, vacuole of the host protoplast. Scale bars: 5 µm (A, D), 10 µm (C, F, G), 20 µm (I). (TIF) [file ppat.1002643.s006.tif]

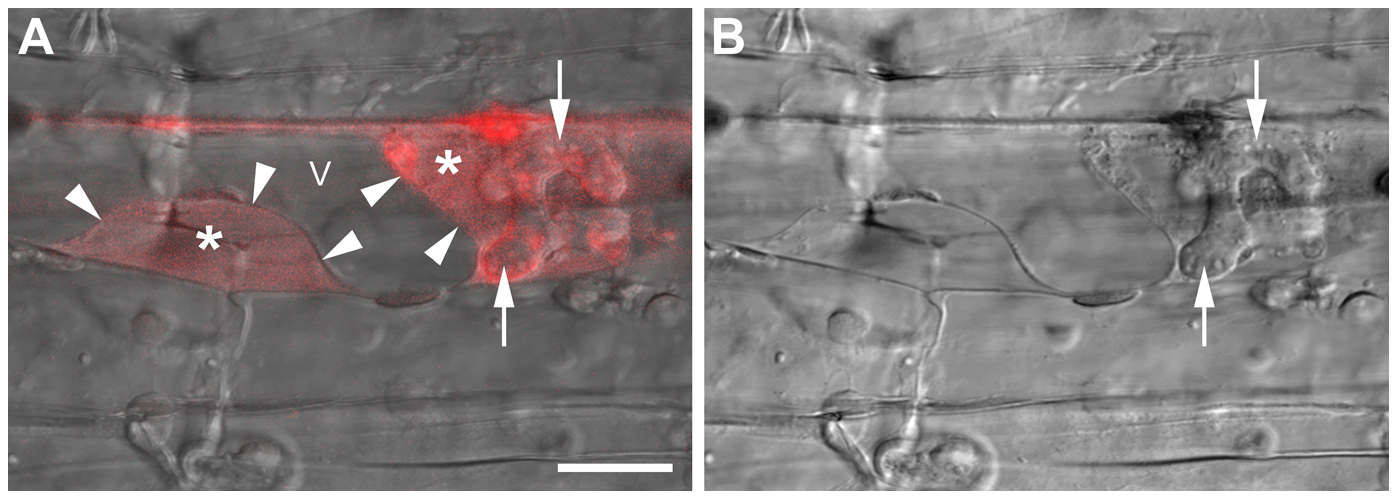

Supplement: Figure S7 — Transformant biotrophic hyphae expressing CHEC89:mRFP viewed with confocal laser scanning microscopy. Epidermal cell infected by a biotrophic hypha (arrows) showing fluorescence in the apoplastic space (*) enlarged by plasmolysis. Arrowheads demarcate the host plasma membrane. V, vacuole of the host protoplast. Scale bar: 10 µm. See also Fig. 4E. (A) identical to Fig. 4E. (B) corresponding brightfield image. (TIF) [file ppat.1002643.s007.tif]

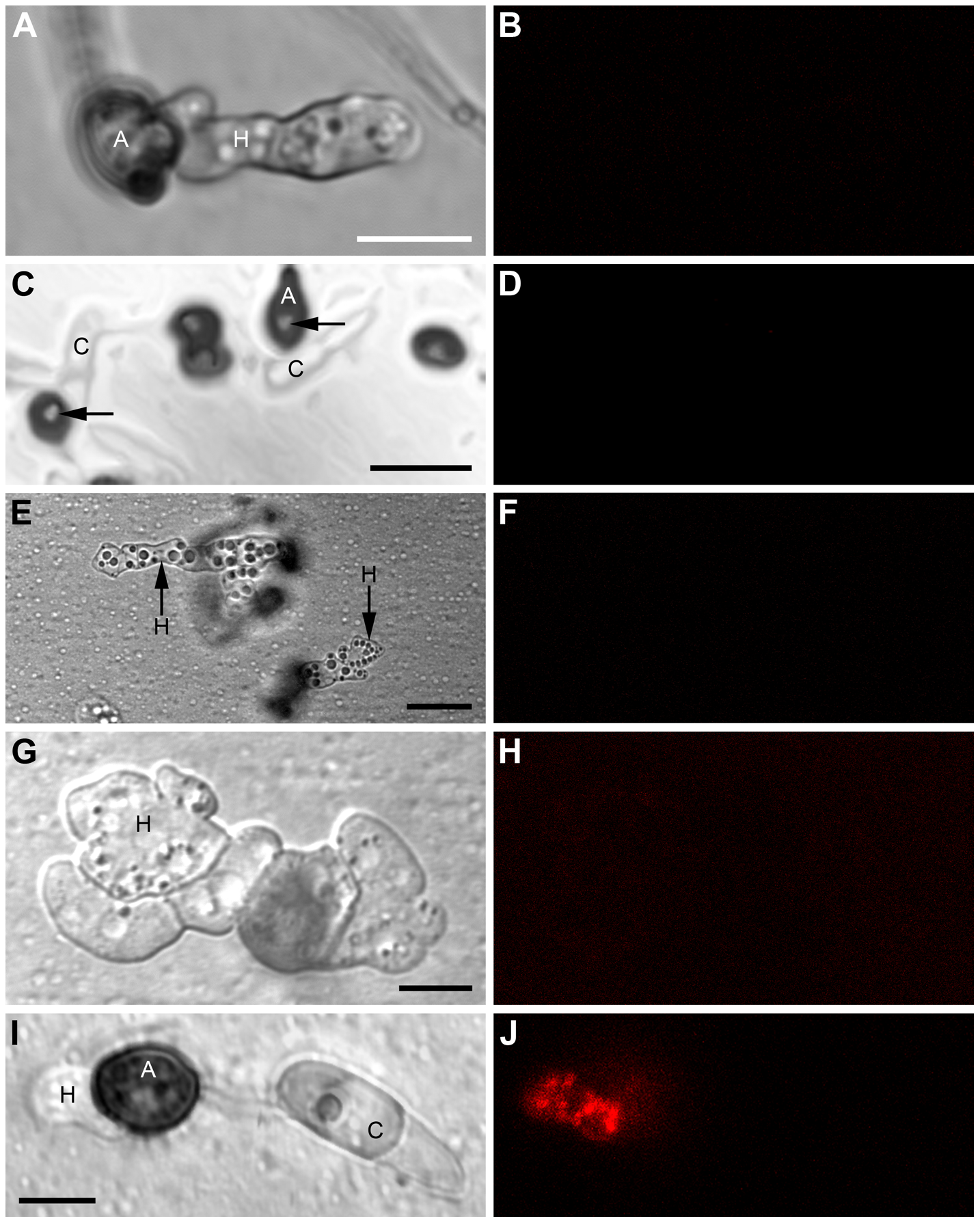

Supplement: Figure S8 — Most ChEC:mRFP fusion proteins are plant-induced and not detectable during penetration of an artificial substratum. Transformants expressing the wave 2 effectors ChEC36:mRFP (A, B) and ChEC6:mRFP (C, D; focus on appressorial penetration pores (arrows) or the wave 3 effectors ChEC89:mRFP (E, F), ChEC34:mRFP (G, H) or ChEC13:mRFP (I, J) were inoculated onto cellophane membranes. (A, C, E, G, I) Bright field images. (B, D, F, H, J) Maximum fluorescence intensity projections. Scale bars: 10 µm (C, E) and 5 µm (A, G, I). A, appressorium. C, conidia. H, pseudo biotrophic hyphae growing inside cellophane. (TIF) [file ppat.1002643.s008.tif]

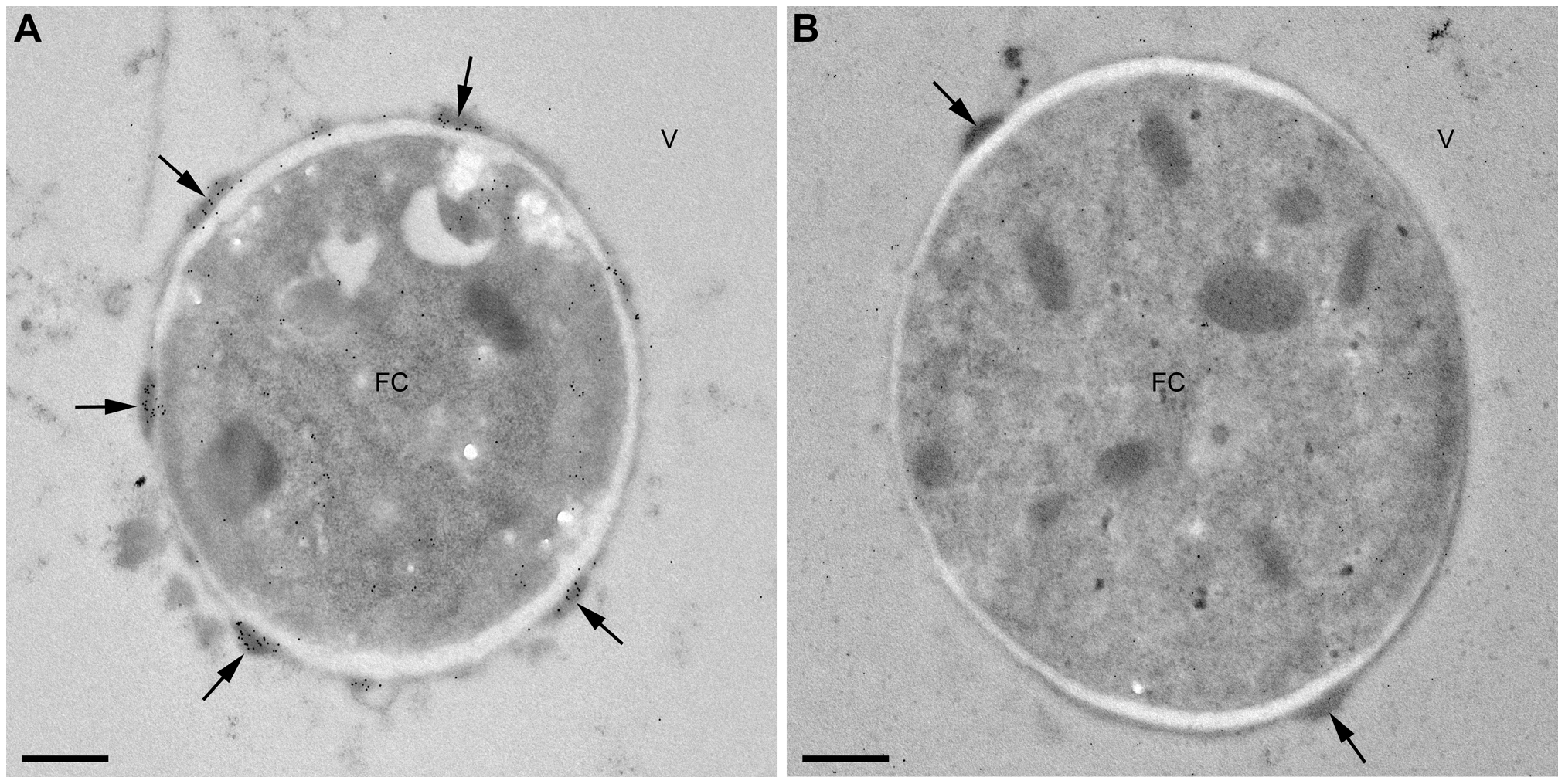

Supplement: Figure S9 — Transmission electron microscopy immunogold detection of ChEC34:mRFP (wave 3 effector). (A) Interfacial bodies of transformant biotrophic hyphae expressing ChEC34:mRFP are labelled. (B) Interfacial bodies of wild-type biotrophic hyphae are unlabelled. Black arrows, interfacial bodies. Scale bar: 500 nm. (TIF) [file ppat.1002643.s009.tif]

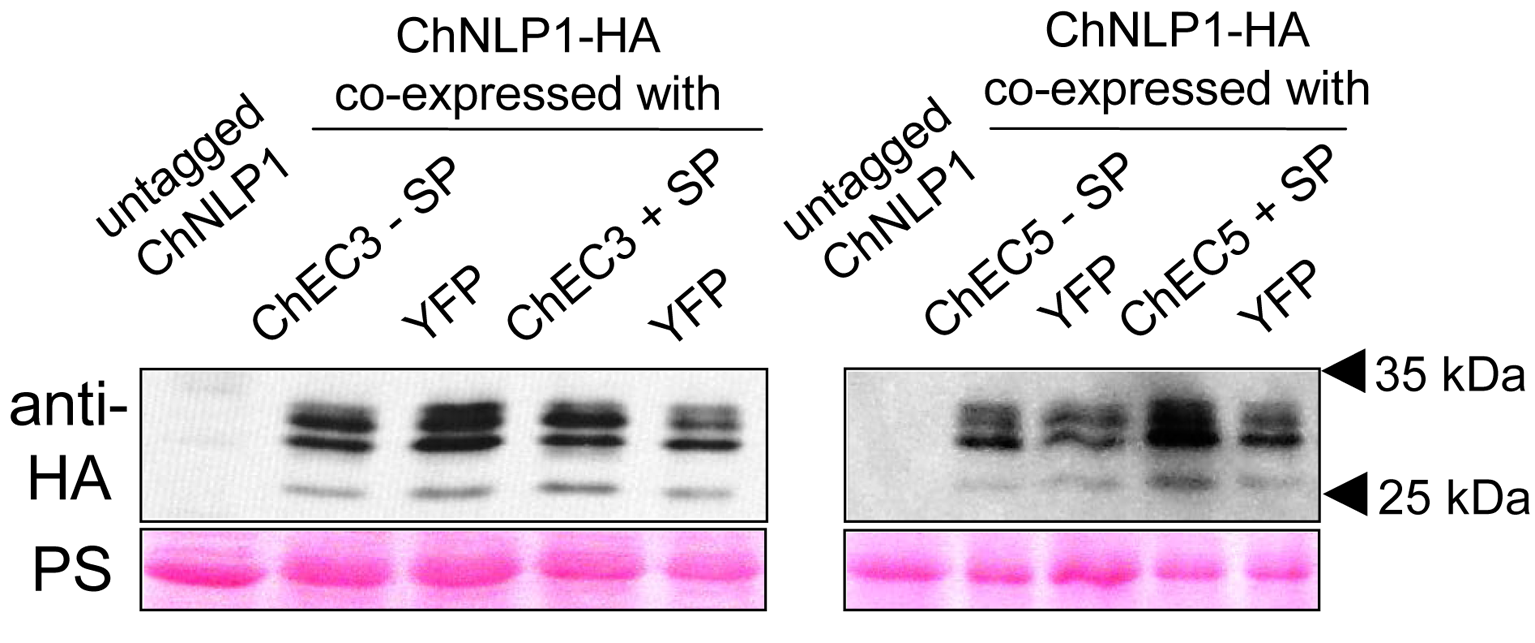

Supplement: Figure S10 — ChNLP1 expression levels are not affected by co-expression of ChECs. ChNLP1 was cloned into a plant expression vector providing a C-terminal translational fusion with a hemagglutinin (HA) tag (ChNLP1-HA). Similar to untagged ChNLP1, ChNLP1-HA was able to induce necrosis, which was found to be suppressable upon ChEC co-expression. Before onset of visible necrotic symptoms (three days after infiltration), eight leaf discs from different sites expressing ChEC/cell death inducer were pooled, likewise their corresponding sites expressing YFP/cell death inducer. ChNLP1-HA protein levels in ChEC3/ChNLP1-HA or ChEC5/ChNLP1-HA pools were compared to those of the corresponding YFP/ChNLP1-HA pools by Western blot analysis. ChEC3 and ChEC5 were expressed either with (+SP) or without (−SP) their signal peptides. Using an anti-HA antibody, full-length ChNLP1-HA (30 kDa expected molecular mass) could be detected, as well as three additional bands between 25 and 30 kDa, indicating partial protein cleavage had occurred. There was no major difference in band intensities between ChEC- and YFP-expressing infiltration site pairs, suggesting that co-expression of ChECs, with or without signal peptide, has no impact on ChNLP protein level per se. PS, Ponceau red stain. (TIF) [file ppat.1002643.s010.tif]
