# Supplementary material for: Sequential Delivery of Host-Induced Virulence Effectors by Appressoria and Intracellular Hyphae of the Phytopathogen Colletotrichum higginsianum
Source: PLoS Pathog. 2012 Apr 5;8(4):e1002643. doi: 10.1371/journal.ppat.1002643 (PMC3320591; doi:10.1371/journal.ppat.1002643)
Supplement: Table S1 — Inventory of biotrophy-associated Colletotrichum higginsianum effector candidates (ChECs). For any ChEC identified, the table lists (a) ENA accession numbers, (b) identifiers refering to the C. higginsianum genome annotated by the Broad institute, (c) protein length, (d) number of cysteines, (e) numbers of homolgues in C. graminicola and (f) predicted motifs or homologies to known genes. (DOC) [file ppat.1002643.s011.doc]

**Supplementary Table 1: Inventory of biotrophy-associated *Colletotrichum higginsianum* effector candidates (ChECs)**

| ChEC name^1)^ | Accession numbers | Gene ID or supercontig (sc) ID^2)^ | Protein length w/o signal peptide predicted from EST contig (aa) | Number of cysteines | % Cysteine residues^3)^ | Homologs in *Colletotrichum graminicola* | Predicted protein motifs / homologies to known effectors^4)^ |  |
| --- | --- | --- | --- | --- | --- | --- | --- | --- |
| ChEC1 | HE651154 | CH063_10826 and CH063_13516 | **44** | 6 | 13,6 | 1 | Identified in our previous study (Kleemann *et al*., 2008) |  |
| ChEC2 | HE651155 | CH063_00152 | **185** | 12 | 6,5 | 1 | dito |  |
| ChEC3 | HE651156 | CH063_04815 | **47** | 2 | 4,3 | 0 | *Colletotrichum gloeosporioides* DN3 (Stephenson *et al*., 2000) |  |
| ChEC3-1 | HE651157 | CH063_04815 | **21** | 0 | 0,0 |  | dito |  |
| ChEC3a | HE651158 | CH063_11733 | **54** | 2 | 3,7 | 0 | dito |  |
| ChEC4 | HE651159 | CH063_13531 | **91** | 0 | 0,0 | 1 | NLS |  |
| ChEC5 | HE651160 | CH063_10072 | **120** | 4 | 3,3 | 3 | Cerato-platanin (IPR010829), *Magnaporthe grisea* MSP1 (Jeong *et al*., 2007) |  |
| ChEC6 | HE651161 | CH063_01084 | **71** | 0 | 0,0 | 1 |  |  |
| ChEC7 | HE651162 | CH063_12827 | **69** | 8 | 11,6 | 1 |  |  |
| ChEC8 | HE651163 | CH063_16106 | **102** | 1 | 1,0 | 1 |  |  |
| ChEC9 | HE651164 | CH063_14473 | **283** | 0 | 0,0 | 1 | NLS |  |
| ChEC10 | HE651165 | absent | **52** | 0 | 0,0 | 0 |  |  |
| ChEC11 | HE651166 | sc 2211: 5501 + | **28** | 6 | 21,4 | 0 |  |  |
| ChEC12 | HE651167 | **CH063_03446** | **82** | 6 | 7,3 | 0 |  |  |
| ChEC12a | HE651199 | CH063_05280 | **81** | 6 | 7,4 | 0 |  |  |
| ChEC13 | HE651168 | sc 3747: 4391 - | **135** | 2 | 1,5 | 0 |  |  |
| ChEC14 | HE651169 | CH063_07863 | **85** | 8 | 9,4 | 0 |  |  |
| ChEC14-1 | HE651170 | CH063_07863 | **33** | 0 | 0,0 |  |  |  |
| ChEC15 | HE651171 | sc 7549: 549 - | **42** | 2 | 4,8 | 0 |  |  |
| ChEC16 | HE651172 | CH063_10447 | **62** | 5 | 8,1 | 0 |  |  |
| ChEC17 | HE651173 | CH063_07414 | **45** | 4 | 8,9 | 1 |  |  |
| ChEC18 | HE651174 | sc 4591: 2630 - | **50** | 5 | 10,0 | 0 |  |  |
| ChEC19 | HE651175 | CH063_03458 | **100** | 0 | 0,0 | 0 |  |  |
| ChEC20 | HE651176 | CH063_13836 | **92** | 6 | 6,5 | 0 |  |  |
| ChEC21 | HE651177 | CH063_03717 | **119** | 8 | 6,7 | 0 |  |  |
| ChEC21a | HE651178 | sc 1057: 8122 - | **89** | 0 | 0,0 | 0 |  |  |
| ChEC22 | HE651179 | sc 698: 3297 + | **96** | 6 | 6,3 | 1 |  |  |
| ChEC23 | HE651180 | CH063_01399 | **68** | 4 | 5,9 | 0 |  |  |
| ChEC24 | HE651181 | sc 729: 7908 - | **42** | 0 | 0,0 | 0 |  |  |
| ChEC25 | HE651182 | sc 285: 9749 - | **41** | 0 | 0,0 | 1 |  |  |
| ChEC26 | HE651183 | CH063_00049 | **44** | 3 | 6,8 | 0 |  |  |
| ChEC26-1 | HE651184 | CH063_00049 | **18** | 0 | 0,0 |  |  |  |
| ChEC26-2 | HE651185 | CH063_00049 | **8** | 0 | 0,0 |  |  |  |
| ChEC27 | HE651186 | sc 4616: 1482 - | **27** | 2 | 7,4 | 0 |  |  |
| ChEC28 | HE651187 | sc 416: 5521 + | **288** | 10 | 3,5 | 0 |  |  |
| ChEC29 | HE651188 | CH063_07433 | **78** | 6 | 7,7 | 0 |  |  |
| ChEC30 | HE651189 | CH063_07266 | **78** | 4 | 5,1 | 1 |  |  |
| ChEC31 | HE651190 | sc 6782:2356 - | **29** | 2 | 6,9 | 0 |  |  |
| ChEC32 | HE651191 | CH063_10904 | **181** | 0 | 0,0 | 1 |  |  |
| ChEC33 | HE651192 | sc 6978: 1695 - | **31** | 6 | 19,4 | 0 |  |  |
| ChEC34 | HE651193 | CH063_02634 | **66** | 7 | 10,6 | 0 |  |  |
| ChEC35 | HE651194 | sc 6436: 1168 - | **108** | 0 | 0,0 | 1 | Enterotoxin, A chain (IPR001144) |  |
| ChEC36 | HE651195 | CH063_09969 | **212** | 6 | 5,3 | 0 | *Fusarium oxysporum* f.sp. *lycopersici* Six6 (Lievens *et al*., 2009) |  |
| ChEC36-1 | HE651196 | CH063_09969 | **198** | 7 | 3,5 |  | dito |  |
| ChEC36-2 | HE651197 | CH063_09969 | **199** | 7 | 3,5 |  | dito |  |
| ChEC37 | HE651198 | sc 390: 7151 - | **28** | 1 | 3,6 | 0 |  |  |
| ChEC38 | HE651200 | sc 843:7339 - | **33** | 8 | 24,2 | 0 |  |  |
| ChEC39 | HE651201 | CH063_11816 | **145** | 6 | 4,1 | 0 |  |  |
| ChEC40 | HE651202 | CH063_13188 and CH063_15487 | **44** | 8 | 18,2 | 0 |  |  |
| ChEC41 | HE651203 | sc 698: 2612 - | **101** | 3 | 3,0 | 1 |  |  |
| ChEC42 | HE651204 | CH063_01004 | **73** | 8 | 11,0 | 0 |  |  |
| ChEC43 | HE651205 | CH063_09679. | **100** | 6 | 6,0 | 1 |  |  |
| ChEC44 | HE651206 | **CH063_10652** | **47** | 6 | 12,8 | 0 |  |  |
| ChEC45 | HE651207 | sc 600: 5466 + | **100** | 2 | 2,0 | 0 |  |  |
| ChEC46 | HE651208 | sc 858: 5121 - | **27** | 0 | 0,0 | 0 |  |  |
| ChEC47 | HE651209 | sc 1142: 5688 - | **68** | 2 | 2,9 | 0 |  |  |
| ChEC48 | HE651210 | sc 2520: 5438 + | **73** | 5 | 6,8 | 0 |  |  |
| ChEC49 | HE651211 | sc 889: 2484 + | **37** | 0 | 0,0 | 0 |  |  |
| ChEC50 | HE651212 | sc 1324: 3219 - | **36** | 1 | 2,8 | 1 |  |  |
| ChEC51 | HE651213 | CH063_09252 | **83** | 0 | 0,0 | 0 |  |  |
| ChEC51a | HE651214 | absent | **181** | 0 | 0,0 | 0 |  |  |
| ChEC52 | HE651215 | absent | **63** | 3 | 4,8 | 1 |  |  |
| ChEC53 | HE651216 | sc 2531: 4987 - | **60** | 1 | 1,7 | 1 |  |  |
| ChEC54 | HE651217 | sc 881: 8053 - | **56** | 1 | 1,8 | 0 |  |  |
| ChEC55 | HE651218 | sc 5543: 566 + | **56** | 0 | 0,0 | 0 |  |  |
| ChEC56 | HE651219 | CH063_08586 | **75** | 8 | 10,7 | 0 |  |  |
| ChEC57 | HE651220 | absent | **45** | 0 | 0,0 | 1 |  |  |
| ChEC58 | HE651221 | sc 622: 5668 - | **24** | 0 | 0,0 | 0 |  |  |
| ChEC59 | HE651222 | sc 1573: 5914 - | **32** | 1 | 3,1 | 0 |  |  |
| ChEC60 | HE651223 | sc 3257: 2769 - | **45** | 2 | 4,4 | 0 |  |  |
| ChEC61 | HE651224 | CH063_09376 | **122** | 7 | 5,7 | 0 |  |  |
| ChEC62 | HE651225 | sc 2373: 1447 + | **63** | 2 | 3,2 | 0 |  |  |
| ChEC63 | HE651226 | sc 8485: 1350 + | **46** | 1 | 2,2 | 0 |  |  |
| ChEC64 | HE651227 | sc 2123: 5083 + | **36** | 2 | 5,6 | 0 |  |  |
| ChEC65 | HE651228 | CH063_07188 | **36** | 4 | 11,1 | 2 |  |  |
| ChEC66 | HE651229 | sc 3813: 572 - | **34** | 0 | 0,0 | 0 |  |  |
| ChEC67 | HE651230 | sc 3909: 1592 - | **33** | 1 | 3,0 | 0 |  |  |
| ChEC68 | HE651231 | sc 7196: 319 - | **35** | 1 | 2,9 | 0 |  |  |
| ChEC69 | HE651232 | CH063_05876 | **28** | 1 | 3,6 | 0 |  |  |
| ChEC70 | HE651233 | sc 10165: 508 - | **46** | 2 | 4,3 | 0 |  |  |
| ChEC71 | HE651234 | sc 6672: 2133 + | **21** | 0 | 0,0 | 1 |  |  |
| ChEC72 | HE651235 | sc 6709: 1347 + | **21** | 0 | 0,0 | 0 |  |  |
| ChEC73 | HE651236 | sc 916: 7360 + | **23** | 2 | 8,7 | 0 |  |  |
| ChEC74 | HE651237 | sc 7289: 1953 + | **49** | 1 | 2,0 | 1 |  |  |
| ChEC75 | HE651238 | sc 18: 11355 - | **22** | 0 | 0,0 | 0 |  |  |
| ChEC76 | HE651239 | sc 1636: 4520 + | **48** | 1 | 2,1 | 0 |  |  |
| ChEC77 | HE651240 | sc 5399: 1433 + | **43** | 2 | 4,7 | 0 |  |  |
| ChEC78 | HE651241 | sc 1571: 5196 - | **26** | 0 | 0,0 | 0 |  |  |
| ChEC79 | HE651242 | sc 5937: 2379 + | **21** | 0 | 0,0 | 0 |  |  |
| ChEC80 | HE651243 | CH063_06824 | **82** | 6 | 7,3 | 2 | Cyanovirin-N (IPR011058) |  |
| ChEC81 | HE651244 | CH063_14530 | **74** | 1 | 1,4 | 0 |  |  |
| ChEC82 | HE651245 | absent | **42** | 2 | 4,8 | 1 |  |  |
| ChEC83 | HE651246 | sc 3891: 826 - | **33** | 0 | 0,0 | 0 |  |  |
| ChEC84 | HE651247 | sc 1468: 7357 - | **33** | 0 | 0,0 | 0 |  |  |
| ChEC85 | HE651248 | CH063_05657 | **69** | 5 | 7,2 | 0 |  |  |
| ChEC86 | HE651249 | CH063_02444 | **108** | 6 | 5,6 | 0 |  |  |
| ChEC87 | HE651250 | CH063_15142 | **47** | 6 | 12,8 | 2 |  |  |
| ChEC88 | HE651251 | CH063_06971 | **78** | 10 | 12,8 | 2 | *Magnaporthe grisea* BAS3 (Mosquera *et al.*, 2009) |  |
| ChEC89 | HE651252 | CH063_02153 | **64** | 4 | 6,3 | 0 |  |  |
| ChEC90 | HE651253 | CH063_13023 | **152** | 6 | 3,9 | 2 | *Colletotrichum lindemuthianum* CIH1, Peptidoglycan-binding lysin domain (IPR018392) |  |
| ChEC90a | HE651254 | CH063_04445 | **105** | 6 | 5,7 | 3 | ditto |  |
| ChEC91 | HE651255 | CH063_11667 | **128** | 4 | 3,1 | 1 | *Ophiostoma ulmi* hypersensitive response-inducing protein (ABK76310) |  |
| ^1)^ Paralogs appearing in this table are indicated by "a". Putative splice variants are indicated by hyphenated numbers. Splice variants of ChEC36 were found by cloning the founder sequence from cDNA of infected plant material and thus are experimentally verified. | | | | | | | | |
| ^2)^ Identifiers refer to the genome assembly and annotation available at <http://www.broadinstitute.org/annotation/genome/colletotrichum_group.3/MultiHome.html>.  If ChECs were not called as genes, the supercontig coordinates (bp, strand) of their N-termini are given. | | | | | | | | |
| ^3)^ Number of cysteines refers to the number of amino acids after signal peptide cleavage. | | | | | | | | |
| ^4)^ In case of identified domains, the InterproScan ID is given | | | | | | | | |
| The coding sequences predicted from these gene models differ from those predicted from the EST contig, e.g. because of (a) single amino acid mismatches caused by sequencing errors, (b) usage of alternative start codons, (c) differently used reading frames and/or wrongly predicted intron-exon structure causing frameshifts, premature stop codons or indels or (d) gene models have been fused by mistake to downstream genes during the automatic annotation of the genome. | | | | | | | | |
| Criteria for considering ChECs as carrying a nuclear localization signal (NLS): NucPred score of greater than 0.2, WoLFPSORT prediction "nuclear" or "cytosolic-nuclear" and predicted by PredictNLS were considered to spend some time in the nucleus. | | | | | | | | |
| Absent in the assembly of the Broad Insitute, but present in an in-house assembly available at <http://www.mpiz-koeln.mpg.de/english/research/pmi-dpt/Fungal_genomes/Colletotrichum/index.html> | | | | | | | | |
| Absent from all current assemblies of the *C. higginsianum* genome. However, the presence of orthologues of ChEC57 and ChEC82 in *C. graminicola* suggest these are genuine *C. higginsianum* genes. ChEC10 was experimentally confirmed to be present in the *C. higginsianum* genome by PCR amplification of the gene from genomic DNA and sequencing the amplicon. | | | | | | | | |
| ChEC7 and ChEC10 contain remnants of retrotransposons in their UTRs | | | | | | | | |
