# Supplementary material for: Sequential Delivery of Host-Induced Virulence Effectors by Appressoria and Intracellular Hyphae of the Phytopathogen Colletotrichum higginsianum
Source: PLoS Pathog. 2012 Apr 5;8(4):e1002643. doi: 10.1371/journal.ppat.1002643 (PMC3320591; doi:10.1371/journal.ppat.1002643)
Supplement: Table S2 — Redundancy of ESTs from plant-penetrating appressoria as a measure for gene expression level: a survey of the top 30 contigs containing the highest numbers of ESTs from plant-penetrating appressoria. The table lists (a) number of ESTs per contig, (b) information on the nearest informative homologue given by BLAST, (c) the presence of a signal peptide, (d) ChEC IDs. (DOC) [file ppat.1002643.s012.doc]

**Supplementary Table 2: Redundancy of ESTs from plant-penetrating appressoria as a measure for gene expression level: list of top 30 *in planta* EST contigs ranked in descending order of absolute and relative numbers of ESTs from plant-penetrating appressoria^1)^**.

| No. PEN APP ESTs | Nearest informative homologue given by BLAST | | | | Secreted? | ChEC ID | Remarks |
| --- | --- | --- | --- | --- | --- | --- | --- |
|  | Description | Species | Accession No. | Expect value |  |  |  |
| 753 | - | *-* | - | - | Y | ChEC6 | - |
| 481 | Retrotransposon *CgT1* | *Colletotrichum gloeosporioides* | L76169 | 2e-51 | Y | ChEC7 | Hybrid transcript with retrotransposon remnant |
| 310 | - | *-* | - | - | Y | ChEC4 | Contains a predicted nuclear localization signal |
| 257 | Conserved hypothetical protein | *Sordaria macrospora* | CBI59290 | 5e-39 | N | - | - |
| 238 | - | *-* | - | - | Y | ChEC8 | - |
| 231 | Hypothetical secreted protein | *Colletotrichum higginsianum* | CAP17696 | 3e-16 | Y | - | Identified in our previous study (Kleemann *et al*., 2008) |
| 215 | - | *-* | - | - | N | - | - |
| 186 | *CgDN3* | *Colletotrichum gloeosporioides* | AAB92221 | 3e-9 | Y | ChEC3 | Presumed effector of *Colletotrichum gloeosporioides* |
| 182 | Hypothetical secreted protein | *Colletotrichum graminicola* | CAQ16226 | 4e-06 | Y | - | - |
| 167 | - | *-* | - | - | N | - |  |
| 164 | - | *-* | - | - | Y | ChEC9 | Contains a predicted nuclear localization signal |
| 150 | - | *-* | - | - | N | - | - |
| 130 | - | *-* | - | - | N | - | - |
| 118 | Cyclophilin | *Magnaporthe grisea* | AAG13968 | 2e-62 | N | - | - |
| 113 | Hypothetical protein | *Podospora anserina* | XP_001903723 | 3e-14 | Y | - | - |
| 94 | - | *-* | - | - | N | - | - |
| 89 | Retrotransposon *Ccret2* | *Colletotrichum cereale* | DQ663512 | 2e-06 | Y | ChEC10 | Hybrid transcript with retrotransposon remnant, gene absent from genome assembly |
| 83 | - | *-* | - | - | Y | ChEC34 | - |
| 80 | Cu/Zn superoxide dismutase | *Verticillium albo-atrum* | EEY18399 | 5e-46 | Y | - | Gene located upstream of ChEC3 |
| 74 | - | *-* | - | - | N | - | - |
| 71 | Hypothetical protein | *Magnaporthe grisea* | XP_368940 | 1e-05 | Y | - | - |
| 67 | - | *-* | - | - | Y | ChEC11 | - |
| 67 | DNA transposase | *Ophiostoma novo-ulmi* | ABG26269 | 3e-09 | N | - | - |
| 67 | - | *-* | - | - | Y | ChEC12 | - |
| 67 | - | *-* | - | - | Y | ChEC13 | - |
| 65 | Hypothetical protein | *Magnaporthe grisea* | XP_001409354 | 2e-08 | Y | - | - |
| 64 | Putative oxidoreductase | *Neosartorya fischeri* | XP_001267508 | 2e-56 | N | - | - |
| 63 | - | *-* | - | - | N | - | - |
| 63 | *CgDN3* | *Colletotrichum gloeosporioides* | AAB92221 | 5e-10 | Y | ChEC3a | Paralogue of ChEC3 |
| 62 | Hypothetical protein | *Magnaporthe grisea* | XP_362922 | 1e-05 | M | - | - |

^1)^ Contigs which were composed of at least 60 % ESTs from plant-penetrating appressoria, but less than 15 % ESTs from the late necrotrophic phase were considered to be enriched for ESTs from plant-penetrating appressoria and unlikely to be constitutive genes expressed at high levels. There was no constraint for ESTs from FACS-isolated biotrophic hyphae. The resulting mean composition of the listed contigs was 92 ± 12 % ESTs from plant-penetrating appressoria, 6 ± 10 % ESTs from FACS-isolated biotrophic hyphae and 2 ± 4 % ESTs from the late necrotrophic phase. The listed contigs account for 13 % of the assembled, high-quality fungal ESTs from plant-penetrating appressoria, suggesting high expression levels.
