# Supplementary material for: Sequential Delivery of Host-Induced Virulence Effectors by Appressoria and Intracellular Hyphae of the Phytopathogen Colletotrichum higginsianum
Source: PLoS Pathog. 2012 Apr 5;8(4):e1002643. doi: 10.1371/journal.ppat.1002643 (PMC3320591; doi:10.1371/journal.ppat.1002643)
Supplement: Text S1 — Supporting materials and methods. Details about (a) employed sequencing and normalization techniques, including assembly statistics, (b) RNA isolation and library preparation, (c) protein domain and motif searches, (d) Sequencing ChEC3 and ChEC3a alleles, (e) RT-PCR, (f) transient expression in N. benthamiana, (g) Western and Southern blot analyses and (h) Cloning procedures and primers used. (DOCX) [file ppat.1002643.s013.docx]

**Text S1**

**Supporting materials and methods**

**Sequencing techniques, strategies to maximize gene discovery, and assembly statistics**

| Biological material (depicted below) | *In planta* ESTs | | | |
| --- | --- | --- | --- | --- |
|  | FACS BH | FACS BH | NECRO | PEN APP |
| Strategy | ’Cold colony picking’ | cDNA normalization | cDNA normalization | Subtraction with cDNA from mock-inoculated epidermis |
| Sequencing method | Sanger | 454 GS FLEX | 454 GS FLEX | 454 GS FLEX |
| Average read length (bp) | 598 | 181 | 158 | 150 |
| Raw reads | 4,770 | 404,299 | 243,154 | 218,811 |
| Contaminating plant sequences  (% of raw reads) | None | 124  (0.03 %) | 25,389  (10.4 %) | 123,978  (56.7 %) |
| High-quality fungal ESTs  (% of raw reads) | 4,027 | 318,604  (78.8 %) | 143,637  (59.1 %) | 38,024  (17.4 %) |
| Contigs | 437 | 35,500  (Co-assembly of 454 ESTs) | | |
| Singletons | 1,023 | 75,681 | | |
| Median coverage | Not determined | 7.9x | | |

FACS BH NECRO PEN APP


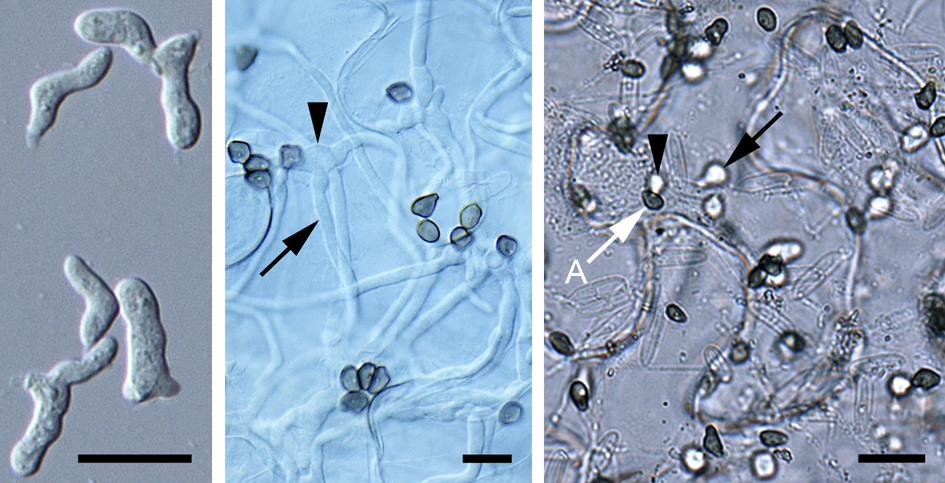


FACS BH: Biotrophic hyphae isolated from infected *Arabidopsis* leaves at 45 hours post-inoculation (hpi) by fluorescence-activated cell sorting.

NECRO: Leaf tissue heavily infested with necrotrophic hyphae (arrow) at 72 hpi, when the leaf is macerated and liquified. Arrowhead: previously biotrophic hypha.

PEN APP: *Arabidopsis* leaf epidermis 22 hpi. Nascent biotrophic hyphae beneath penetrating appressoria (A) are globular structures with high optical refraction (arrowhead). Some appressoria became detached during epidermal peeling, leaving biotrophic hyphae only (arrow). Scale bars, 10 µm.

**RNA isolation, library preparation and EST handling**

RNA from *in vitro* germlings and appressoria was prepared as described previously (Kleemann et al., 2008). RNA from spores, mycelium and infected plant material was extracted using the RNeasy Mini Kit (Qiagen), including DNAse treatment. RNA samples were checked on an Agilent 2100 Bioanalyzer to verify sufficient RNA integrity. Total RNA from epidermal peels containing appressoria with and without nascent biotrophic hyphae were mixed in equal amounts for the cDNA library representing plant-penetrating appressoria. Details for cDNA library preparation, EST sequencing, quality control and filtering are given elsewhere (Kleemann, 2010; Spanu et al., 2010). ESTs from *in planta* infection stages were co-assembled using SeqMan pro v.8 (Lasergene) with default parameters for Roche 454 data. Contigs were mapped and visualized onto a draft assembly of the *C. higginsianum* genome using Blat and GBrowse (Stein et al., 2002) and are available at http://gbrowse.mpiz-koeln.mpg.de/cgi-bin/gbrowse/colletotrichum_higginsianum/.

**’Cold colony picking’ to enrich for non-redundant cDNA clones from biotrophic hyphae isolated by fluorescence-activated cell sorting**

cDNA preparation and cDNA library construction were described previously (Takahara et al. 2009). Transformed *E. coli* were plated onto 22 x 22 cm LB plates containing 50 µg/ml ampicillin, 20 µg/ml X-gal, and 1 mM IPTG. White recombinant colonies were picked with a QPix2 colony picker (Genetix) into 384-well microtiter plates containing LB supplemented with 8 % glycerol and 50 µg/ml ampicillin followed by incubation for 18 h at 37 ºC. Bacterial colonies were then arrayed onto nylon membranes supported on a LB plate using a MicroGrid II robotic spotting tool (BioRobotics) and incubated for 8 h at 37 ºC until colonies were visible. After alkaline denaturation and neutralization, the filters were rinsed in 2x SSC, air dried and UV cross-linked. Filters were treated with proteinase K in 2x SSC for 1 h at 37 ºC followed by gentle removal of bacterial debris. DIG-labelled probes matching the 10 most highly expressed genes (Takahara et al., 2009) were synthesized and used for hybridization according to the manufacturer’s instructions (Roche). Clones which did not give a chemiluminescence signal were re-formatted into a 96-well plate for DNA preparation and sequencing.

**Protein domain and motif search**

Protein domains and motifs were sought by querying the NCBI conserved domain database (Marchler-Bauer et al., 2011), InterProScan (Zdobnov and Apweiler, 2001), MEME (Bailey and Elkan, 1994), NucPred (Brameier et al., 2007), PredictNLS (Cokol et al, 2000) and WolfPSORT (Horton et al., 2007). Sequence alignments and secondary structure predictions were made using ClustalW (Larkin et al., 2007) and PSIPRED (Bryson et al., 2005), respectively. Although not a selection criterion, none of the biotrophy-associated ChECs contained potential glycosyl-phosphatidylinositol-anchors, as determined by Fungal Big-PI Predictor (Eisenhaber et al., 2004).

**Sequencing of *ChEC3* and *ChEC3a* alleles**

Amplification of *ChEC3* and *ChEC3a* genes from different *C. higginsianum* isolates (see table below) was accomplished with primers encompassing the region between signal peptide cleavage site and stop codon. Standard PCR was carried out using Taq DNA polymerase (Amplicon) according to the manufacturer’s instructions. Nucleotide polymorphisms found in an initial screen were confirmed by a second PCR. DNA sequences were determined at the Max-Planck Genome Centre Cologne on Applied Biosystems (Weiterstadt, Germany) Abi Prism 377, 3100 and 3730 sequencers using BigDye-terminator v3.1 chemistry. Premixed reagents were from Applied Biosystems.

| Isolate ID | Isolated from | Country origin | Reference |
| --- | --- | --- | --- |
| IMI 349063A | *Brassica campestris* | Trinidad & Tobago | 1 |
| IMI 349061 | *B. campestris* | Trinidad & Tobago | 1 |
| CH90-M3 | *Matthiolaincana* | Japan | 2 |
| CH93-M1 | *M. incana* | Japan | 2 |
| AR 3-1 | *Raphanus sativus* | Japan | 2 |
| NBRC6182 | *Brassica sp.* | Italy | 2 |
| Abo1-1 | *B. oleracea* | Japan | 2 |
| Abp 3-1 | *B. pekinensis* | Japan | 2 |
| MAFF 305968 | *B. chinensis* | Japan | 2 |
| MAFF 305970 | *B. pekinensis* | Japan | 2 |
| Abr 3-1 | *B. rapa* | Japan | 2 |
| Abcr 1-2 | *B. cernua* | Japan | 2 |
| CH93-M4 | *M. incana* | Japan | 2 |
| CH93-M5 | *M. incana* | Japan | 2 |
| AR 3-5 | *R. sativus* | Japan | 2 |
| Kyoto 337-5 | *B. napus* | Japan | 3 |
| MAFF 305635 | *B. rapa* | Japan | 2 |
| Aba 1-1 | *B. parachinensis* | Japan | 2 |
| Abp 7-1 | *B. pekinensis* | Japan | 2 |
| Abc 3-1 | *B. chinensis* | Japan | 2 |
| Abj 1-2 | *B. japonica* | Japan | 2 |

The fungal isolates were obtained from the following culture collections: LARS, Long Ashton Research Station; MAFF, Japanese Ministry of Agriculture, Fisheries and Food; IMI, International Mycological Institute of the UK; NBRC, Japanese National Institute of Technology and Evaluation, Biological Resource Center; 1, O'Connell *et al*., 2004; 2, Moriwaki *et al*., 1997; 3, Ushimaru *et al*., 2010

**Expression analysis of ChNLPs by RT-PCR**

Total RNA (2.5 µg) was mixed with 50 ng Oligo-dT-primer, heated to 80 °C for 3 min to remove any secondary structures and then quickly chilled on ice. The following components were added (final concentration in a 20 µL-reaction): 1x First strand buffer (Invitrogen), 0.5 mM dNTPs (Roth), 10 mM dithiothreitol (Invitrogen), 0.5 U/µL RNase Inhibitor (Roche) and 10 U/µL SuperScript II (Invitrogen). Reverse transcription was carried out for 5 min at 23 °C, 1 h at 42 °C and 10 min at 50 °C, followed by heat inactivation at 80 °C for 3 min. The resulting cDNAs were diluted 1:50 to 1:100 with TE and represented the cDNA stock that was used for RT-PCR experiments. PCR was carried out using Taq DNA polymerase (Amplicon) according to the manufacturer’s instructions. The *C. higginsianum* alpha-tubulin gene was used to adjust cycle numbers and loading of individual PCR reactions to allow for variation in fungal biomass. Each RT-PCR was performed at least three times with similar results.

**Transient expression in *N. benthamiana***

Recombinant *A. tumefaciens* strains were grown in LB supplemented with appropriate antibiotics: rifampicin, carbenicillin, spectinomycin and streptomycin (all at 50 µg/mL) for constructs in pB7WG2 and bacterial strain C58C1 pGV2260; rifampicin (100 µg/mL), carbenicillin (50 µg/mL), kanamycin (25 µg/mL) and gentamycin (15 µg/mL) for constructs in pXCSG3xHA; rifampicin and kanamycin (both at 50 µg/mL) for an *A. tumefaciens* strain carrying a construct for expression of p19, a viral suppressor of gene silencing (Voinnet *et al*., 2003). All bacterial cultures were grown to stationary phase to maximize transformation efficiency (Marion *et al*., 2008). Bacterial cells were pelleted and resuspended in infiltration buffer (10 mM MgCl_2_, 5 mM MES (pH 5.6) supplemented with 200 µM acetosyringone) before infiltration into the abaxial side of *N. benthamiana* leaves using a needle-less syringe.

Infiltration mixtures containing bacteria harbouring constructs for co-expression of cell death inducers (INF1 and ChNLP1) together with ChECs or YFP as control were mixed according to the table below. Infiltration mixures were kept at room temperature for 2‑4 h before infiltration into fully-expanded leaves of *N. benthamiana* plants. To allow pairwise comparisons, infiltration mixtures containing ChEC/cell death inducer (CDI) constructs and YFP/CDI constructs were infiltrated side-by-side into the same leaf. Plants were incubated in a controlled environment chamber (19 °C/21 °C day/night temperature cycles and 16-h-light/8-h-dark cycles) to which they were adapted at least 24 h before infiltration. Six to eight days after infiltration, infiltration site pairs were inspected in a blinded manner to determine whether the site co-expressing ChECs with CDI showed reduced necrosis compared to the corresponding control site on the same leaf expressing YFP with CDI.

Final OD_600_ of recombinant *A. tumefaciens* strains used in infiltration mixtures.

|  | Final OD_600_ of respective *A. tumefaciens* strains expressing the indicated proteins | | | |
| --- | --- | --- | --- | --- |
| Infiltration mixture | ChEC | YFP | Cell death inducer | p19 |
| ChEC expression | 1.0 | - | 0.1 | 0.5 |
| Corresponding YFP control | - | 1.0 | 0.1 | 0.5 |

**Western blot analyses**

Leaf discs (6 mm diameter) of agroinfiltrated areas of *N. benthamiana* leaves were harvested at 3 dpi, before the onset of visible necrotic symptoms. Eight leaf discs from sites expressing ChEC/CDI were pooled, likewise eight sites expressing YFP/CDI. After grinding in liquid nitrogen, ground tissue was thawed in 1 mL extraction buffer (50 mM Tris pH 8, 50 mM NaCl, 5 mM EDTA acid, 0.01 % Triton, 5 mM DTT) including 1x complete protease inhibitor (Roche). Protein samples boiled for 5 min in 2x SDS sample buffer were subjected to SDS-PAGE on a gel containing 12 % polyacrylamide, followed by electroblotting on a Hybond ECL nitrocellulose membrane (Amersham, GE Healthcare). To monitor protein transfer and loading, the membrane was stained with 0.1 % Ponceau S (Sigma-Aldrich) in 5 % acetic acid, followed by extensive washes in PBS and photographic documentation. For HA detection, commercial antibodies were used (Roche).

**Southern blot analysis**

Extraction of fungal genomic DNA was carried out as described by Huser and co-workers (2009). For Southern blot hybridization, 10 µg of genomic DNA was digested to completion with 10 U of restriction enzyme EcoRV (New England Biolabs, Frankfurt, Germany). After gel-electrophoresis, the DNA was transferred onto Amersham Hybond N++ membrane (GE Healthcare) by alkaline transfer. The membrane was hybridized with a full-length cDNA probe, labelled with digoxigenin (DIG)-dUTP, using the PCR DIG probe synthesis kit (Roche, Mannheim, Germany) according to the manufacturer’s instructions. For characterization of transformants, the membrane was hybridized and washed under high stringency conditions (prehybridization and hybridization at 50 °C, high-stringency wash at 68 °C with 0.1x SSC containing 0.1 % SDS). For detection of homologous sequences in different *Colletotrichum* species and isolates, the membrane was hybridized and washed under low stringency conditions (prehybridization and hybridization at 35 °C, high-stringency wash at 60 °C with 0.5x SSC containing 0.1 % SDS). Assuming 1.1 M Na+ and 50 % formamide, these conditions were calculated to allow 25 % mismatches between probe and target DNA, according to the manufacturer’s instructions (Roche). Probe hybridization was detected using the DIG luminescence detection kit according to the manufacturer’s instructions (Roche). Membranes were stripped afterwards for further hybridizations using alkaline treatment.

**Cloning**

All primers used in this study (see tables below) were designed with the Primer3 program. To clone signal peptide-less versions of ChECs, codons following the predicted signal peptide cleavage site were fused to an artificial start codon. Following TOPO cloning (Invitrogen) and sequence verification, cloned sequences were shuttled *via* Gateway recombination into (i) the binary plant expression vector pB7WG2 providing expression by the CaMV 35S promotor (VIB Gent, Gent university) for the cell death suppression assay, (ii) pXCSG3xHA (Feys et al., 2005) for HA-tagging of ChNLP1, (iii) pXCSG-GFP for C-terminal translational fusions of ChEC4 and ChEC9 to GFP (Feys et al., 2005) or (iv) pEDV6 (Sohn et al., 2007) for the bacterial growth assay. *A. tumefaciens* strain C58C1 pGV2260 was used as the recipient strain for the cell death suppression assay.

**Primers used for qRT-PCR**

| **Name** | **Forward** | **Reverse** | **Amplicon length (bp)** | **Efficiency (%)** |
| --- | --- | --- | --- | --- |
| α tubulin | GAGCGCCCTAACTACGAGAA | CGAAGCAGGACATGGTCATC | 232 | 103 |
| Actin | CCCCAAGTCCAACAGAGAGA | CATCAGGTAGTCGGTCAAGTCA | 238 | 100 |
| ChEC3 | CGCTCTTCCCTTTACAACCA | ATATTCCACGCCCACACATT | 153 | 106 |
| ChEC3a | ACTGTTCCCTTTGCCTCTCA | AGGTTCCACACAGCCACATT | 173 | 110 |
| ChEC6 | CGCCATTCTTGCCATCATT | GAGGACTTGCTTGGGATCAG | 256 | 106 |
| ChEC4 | GGCCAACTCGAAACCAGTAT | TCGCAATGGAAGTGCTTATG | 113 | 97 |
| ChEC7 | ATACTGTCCGGCTTCACGTT | TCTTCCAATTTCGCCTCCTT | 125 | 99 |
| ChEC9 | AACGAGCGGAGTGGACAAG | ATCTCGTCGCGCTCTCTGT | 108 | 102 |
| CHEC89 | TGGAACACCGGCAACTATG | TAAGGGCAGCTCCAGTAGG | 119 | 106 |
| CHEC34 | CCCAACTGCAACTTCTGTCTC | TGAAGGACCGCGACTGAT | 111 | 115 |
| ChEC56 | GCCCCGAAAGCTATGTCTC | GAAAGCGCCTCCGCTAAT | 167 | 109 |
| ChEC51 | CGGGGAACCTGATGAAGTAG | TCCCACCGTATCGAAGTTTT | 218 | 115 |
| ChEC5 | TGGTTTTGCCAGCCATATCT | CCTCGTCACCTTCCTCTCTTC | 150 | 105 |
| CHEC13 | CCGTCATGTCCCTAGCTGTT | GGTAGCGCCGAAGTCAAATA | 185 | 109 |
| CHEC88 | CAACTCCCGGTTTCTAACGA | TCGCAGTCAATAACGCAAAG | 122 | 116 |
| ChEC36 | TTTGTGCCAACAACGAAGTC | ATTGGGTCTGTCCTCCATTG | 106 | 115 |
| CHEC91 | AGTGCCTCATCGCTTTCAAC | AACCGGGCGAGCAATTTA | 329 | 100 |
| ChNLP1 | CACCGTCGGCAACTTGTA | CGAGTCCTTGGGCATGTAGT | 226 | 98 |
| ChToxB | CACCATGAAGTTCACCGCCGCCATC | TCAGCAGAACCCGGCTTTGC | 261 | 123 |

**Other primers used in this study.**

| Primer | Remark | Sequence (5’→3’) |
| --- | --- | --- |
| **Cloning of protein coding sequences** | | |
| ChEC3 ORF fw |  | CACCATGTACTTTACAAACATCTTCG |
| ChEC3 ORF –SP fw | without signal peptide | CACCATGCTCCCTGCCAATAAGCATATAGG |
| ChEC3 ORF rev | with stop codon | TCAACATTTAAACTTTCCACAG |
| ChEC3a ORF fw |  | CACCATGTACGCCACCAAGATCATCT |
| ChEC3a ORF –SP fw | without signal peptide | CACCATGCTCCCTGCTGAAGTGCATAAGG |
| ChEC3a ORF rev | with stop codon | TTAACACTTGACCTTCCCACAA |
| ChEC5 ORF fw |  | CACCATGCAGCTCTCCGGCCTCGTC |
| ChEC5 ORF –SP fw | without signal peptide | CACCATGGTCTCCGTCTCCTACGACACCG |
| ChEC5 ORF rev | with stop codon | CTACAGGCCGCAGGCGTTAAGG |
| ChEC36 ORF fw |  | CACCATGGTGGTCATTCCTCTCTCTCAAGTTG |
| ChEC36 ORF rev | with stop codon | TTAAATAAGCCAAGAAAAGGCATTT |
| CHEC89 ORF fw |  | CACCATGCAGTTCTTCAACGCCATCATCGTTTTCG |
| CHEC89 ORF rev | with stop codon | TTAAGGGCAGCTCCAGTAGG |
| CHEC34 ORF fw |  | CACCATGCGTACCGCTACCGTTACCGCCGCCTTTG |
| CHEC34 ORF rev | with stop codon | TTAGAGGCAGCACGTAAGCATGCACTGGAAG |
| CHEC13 ORF fw |  | CACCATGAAGTTCTTTGCCTCTCTTTTGGCCGTTGC |
| CHEC13 ORF rev | with stop codon | CTAGGACTCCATCTGCACATACCAAGTACC |
| ChEC4 ORF –SP fw | without signal peptide | CACCATGGCTCCCACGGGCGATAACATAAGCACTTCCATTGC |
| ChEC4 ORF rev | without stop codon | CGCCTGGCCAACCTTGCCACCG |
| ChEC9 ORF –SP fw | without signal peptide | CACCATGGCACCTCTCGTCAATCGTTTCGCGAACGATGG |
| ChEC9 ORF rev | without stop codon | GACCCTGCCGTTTCCTGATTTAC |
| ChNLP1 ORF fw |  | CACCATGGCCCCCTCGCTCTTCCGTC |
| ChNLP1 ORF rev | with stop codon | TTACAACGCGGCCTTGCCG |
| ChNLP1 ORF rev oS | without stop codon, for 3xHA tagging | CAACGCGGCCTTGCCG |
| ChNLP3 ORF fw |  | CACCATGCAGTTTTTAACTCTTCTTAGTCTCTTGG |
| ChNLP3 ORF rev | with stop codon | TCACTTCTTGCAAACTGGCTCCTCCGGCACG |
| Chitinase ORF fw |  | CACCATGTCCTTCGCTAAGTTGTCGCTCGCGGCCTTGC |
| Chitinase ORF –SP fw | without signal peptide | CACCATGGCGCCATGCCCCGACAACGCCACTACCGGCATCG |
| Chitinase ORF rev | with stop codon | GTATCCCGCAAAATACGTCGAG |
| **Fluorescent protein tagging of ChECs** | | |
| pChEC36-fw |  | CACCCTGGCTCAACAGTAGTTCCTAATTC |
| ChEC36-rev | without stop codon | AATAAGCCAAGAAAAGGCATTTAG |
| pChEC6-fw |  | TGTGGGTGAGATTAAGGATAGGTTTG |
| ChEC6-rev | without stop codon | GCCGAGGACTTGCTTGGGATCAGC |
| pChEC3-fw |  | CACCACTAACATGCTCTCACGTAGGAACT |
| ChEC3-rev | without stop codon | ACATTTAAACTTTCCACAGTGTGCT |
| pCHEC89-fw |  | CACCTACACTGTAGTAAGCCCACTGTTAC |
| CHEC89-rev | without stop codon | AGGGCACTAGAGATCAACCCATTGTTA |
| pCHEC34-fw |  | CACCTTCTCTTGCTAACAGACCAGAAGTA |
| CHEC34-rev | without stop codon | GAGGCAGCACGTAAGCATGCACTGG |
| pCHEC13-fw |  | CACCGAGTGATGATGGAGGTGAAG |
| CHEC13-rev | without stop codon | GGACTCCATCTGCACATACC |
| **ChNLP transcript detection** | | |
| OligodT primer |  | CGGCCGCGAATTCACTAGTGTTTTTTTTTTTTTTTTTT |
| ChNLP1 fw |  | ATGGCCCCCTCGCTCTTCCGTC |
| ChNLP1 rev |  | TTACAACGCGGCCTTGCCG |
| ChNLP2 fw |  | AGGTTCAACCCACTTCTCCATGTC |
| ChNLP2 rev |  | TCGTTTAGAGCACTCTGCATCGTC |
| ChNLP3 fw |  | TACTGGCAGTCTAGGTGGTGGTTTG |
| ChNLP3 rev |  | GCACTTGGCTCGCATAATAATTGG |
| ChNLP4 fw |  | ATACCCTCCTGAGGCGTAAAGCTC |
| ChNLP4 rev |  | AAAACCACTTCCCAAGGTGATATCTC |
| ChNLP5 fw |  | GGCACTACTACCTGACTGTCGTCGT |
| ChNLP5 rev |  | GTTCGTATTGGATGCCGTTGAACT |
| ChNLP6 fw |  | TGAGGCCACTCTCAAGTTCATTACC |
| ChNLP6 rev |  | CGGTTGTCCAACAAAAATGAGACAC |
| Tubulin fw |  | GCCCTATTCTCGCTCGTCTTCC |
| Tubulin rev |  | GGGCTCCAAATCGCAGTAAATG |
| **Southern probes** |  |  |
| ChEC1 fw |  | GATCAAACACAATCGCCAAAAATGAAGTC |
| ChEC1 rev |  | TGTTCCTTTGATGCCATGTTTG |
| ChEC2 fw |  | ATGCTCTACTCCAAAATCCTCATCGCCGCC |
| ChEC2 rev |  | ATCATCTAAGCCTGGTTGACG |
| ChEC3 fw |  | GATACGCAAATCCTTAGTATTAAAGAAGC |
| ChEC3 rev |  | ATCTTTGCATCACTTGTATTGC |
| Calpain fw |  | GCCGCCCTCTTCTCCGTCTTCTG |
| Calpain rev |  | ATGGTGTGGGCGGAGTGGTTATCG |
| **Miscellaneous primers** |  |  |
| ChEC10 fw | To verify presence in the genome | TGATTGCTGAAGAGCTATGGAAGG |
| ChEC10 rev | To verify presence in the genome | CTGACTTCCCATCTCCACAATTC |
| ChEC3 seq fw | Amplification and sequencing of alleles | CTCCCTGCCAATAAGCATATAGG |
| ChEC3 seq rev | Amplification and sequencing of alleles | TCAACATTTAAACTTTCCACAGTGTGC |
| ChEC3a seq fw | Amplification and sequencing of alleles | CTCCCTGCTGAAGTGCATAAGG |
| ChEC3a seq rev | Amplification and sequencing of alleles | TTAACACTTGACCTTCCCACAATG |

**References specific to Text S1**

Bailey, T.L. and Elkan, C. (1994). Fitting a mixture model by expectation maximization to discover motifs in biopolymers. In Proceedings of the 2^nd^ International Conference on Intelligent Systems for Molecular Biology, (Menlo Park, California: AAAI Press), pp. 28-36.

Brameier, M., Krings, A., MacCallum, R.M. (2007). NucPred-predicting nuclear localization of proteins. Bioinformatics *23*, 1159-1160.

Bryson, K., McGuffin, L.J., Marsden, R.L., Ward, J.J., Sodhi, J.S., and Jones, D.T. (2005). Protein structure prediction servers at University College London. Nucleic Acids Res. *33* W36-38.

Cokol, M., Nair, R., Rost, B. (2000). Finding nuclear localization signals. EMBO Rep. *1*, 411-415.

Eisenhaber, B., Schneider, G., Wildpaner, M. and Eisenhaber, F. (2004). A sensitive predictor for potential GPI lipid modification sites in fungal protein sequences and its application to genome-wide studies for *Aspergillus nidulans*, *Candida albicans*, *Neurospora crassa, Saccharomyces cerevisiae* and *Schizosaccharomyces pombe*. J. Mol. Biol. *337*, 243-253.

Feys, B. J., Wiermer, M., Bhat, R. A., Moisan, L. J., Medina-Escobar, N., Neu, C., Cabral, A. and Parker, J. E. (2005). *Arabidopsis* senescence-associated gene101 stabilizes and signals within an enhanced disease susceptibility1 complex in plant innate immunity. Plant Cell. *17*, 2601-2613.

Horton, P., Park, K.-J., Obayashi, T., Fujita, N., Harada, H., Adams-Collier, C.J., and Kenta, N. (2007). WoLF PSORT: Protein Localization Predictor. Nucleic Acids Res. *35* (suppl 2) W585-W587*.*

Larkin, M.A., Blackshields, G., Brown, N.P., Chenna, R., McGettigan, P.A., McWilliam, H., Valentin, F., Wallace, I.M., Wilm, A., Lopez, R., et al .(2007). ClustalW2 and ClustalX version 2. Bioinformatics *23*, 2947-2948.

Marchler-Bauer, A., Lu, S., Anderson, J.B., Chitsaz, F., Derbyshire, M.K., Deweese-Scott, C., Fong, J.H., Geer, L.Y., Geer, R.C., Gonzales, N.R., et al. (2011). CDD: a Conserved Domain Database for the functional annotation of proteins. Nucleic Acids Res. *39*, D225-D229.

Marion, J., Bach, L., Bellec, Y., Meyer, C., Gissot, L., Faure, J.-D. (2008). Systematic analysis of protein subcellular localization and interaction using high-throughput transient transformation of *Arabidopsis* seedlings. Plant J., *56*, 169–179.

Moriwaki, J., Ohkubo, H., Horie, H., Kasuyama, S., and Sato, T. (1997). Genotypic differences between *Colletotrichum higginsianum* and *C. gloeosporioides* based on arbitrary primed PCR analysis. Ann. Phytopathol. Soc. Jpn. *63*, 395-398.

Spanu, P. D. Abbott, J. C., Amselem, J., Burgis, T. A., Soanes, D. M., Stüber, K., Ver Loren van Themaat, E., Brown, J. K. M., Butcher, S. A., Gurr, S. J. et al. (2010). Genome Expansion and Gene Loss in Powdery Mildew Fungi Reveal Tradeoffs in Extreme Parasitism. Science *330*, 1543-1546.

Stein, L. D., Mungall, C., Shu, S., Caudy, M., Mangone, M., Day, A., Nickerson, E., Stajich, J. E., Harris, T. W., Arva, A., Lewis, S. (2002). The generic genome browser: a building block for a model organism system database. Genome Res. *12*, 1599-1610.

Ushimaru, T., Terada H., Tsuboi, K., Kogou Y., Sakaguchi, A., Tsuji, G. and Kubo, Y. (2010). Development of an efficient gene targeting system in *Colletotrichum higginsianum* using a non-homologous end-joining mutant and *Agrobacterium tumefaciens*-mediated gene transfer. Mol. Genet. Genomics *284*, 357-371.

Voinnet, O., Rivas, S., Mestre, P., Baulcombe, D. (2003). An enhanced transient expression system in plants based on suppression of gene silencing by the p19 protein of tomato bushy stunt virus. Plant J., *33*, 949–956.

Zdobnov, E.M. and Apweiler, R. (2001). InterProScan - an integration platform for the signature-recognition methods in InterPro. Bioinformatics *17*, 847-848.
